# The HOXC10/NOD1/ERK axis drives osteolytic bone metastasis of pan-*KRAS*-mutant lung cancer

**DOI:** 10.1038/s41413-024-00350-8

**Published:** 2024-08-27

**Authors:** Kun Li, Bo Yang, Yingying Du, Yi Ding, Shihui Shen, Zhengwang Sun, Yun Liu, Yuhan Wang, Siyuan Cao, Wenjie Ren, Xiangyu Wang, Mengjuan Li, Yunpeng Zhang, Juan Wu, Wei Zheng, Wangjun Yan, Lei Li

**Affiliations:** 1 Department of Musculoskeletal Oncology, Fudan University Shanghai Cancer Center, Shanghai, 200032 China; 2 Health Science Center, East China Normal University, Shanghai, 200241 China; 3 Chongqing Key Laboratory of Precision Optics, Chongqing Institute of East China Normal University, Chongqing, 401120 China; 4 School of Life Sciences, East China Normal University, Shanghai, 200241 China; 5 Joint Center for Translational Medicine, Shanghai Fifth People’s Hospital, Fudan University and School of Life Science, East China Normal University, Shanghai, 200240 China; 6 Department of Pharmacy The General Hospital of Western Theater Command, Chengdu, 610083 China; 7 Orthopaedic Department of Shanghai Jiao Tong University Affiliated Sixth People’s Hospital, Shanghai, 200233 China; 8 Department of Orthopedics, General Hospital of Western Theater Command, Chengdu, 610000 China; 9 College of Medicine, Southwest Jiaotong University, Chengdu, 610031 P. R. China

**Keywords:** Bone cancer, Cancer

## Abstract

While *KRAS* mutation is the leading cause of low survival rates in lung cancer bone metastasis patients, effective treatments are still lacking. Here, we identified homeobox C10 (HOXC10) as a lynchpin in pan-*KRAS*-mutant lung cancer bone metastasis. Through RNA-seq approach and patient tissue studies, we demonstrated that HOXC10 expression was dramatically increased. Genetic depletion of *HOXC10* preferentially impeded cell proliferation and migration in vitro. The bioluminescence imaging and micro-CT results demonstrated that inhibition of *HOXC10* significantly reduced bone metastasis of *KRAS*-mutant lung cancer in vivo. Mechanistically, the transcription factor HOXC10 activated NOD1/ERK signaling pathway to reprogram epithelial-mesenchymal transition (EMT) and bone microenvironment by activating the *NOD1* promoter. Strikingly, inhibition of *HOXC10* in combination with STAT3 inhibitor was effective against *KRAS*-mutant lung cancer bone metastasis by triggering ferroptosis. Taken together, these findings reveal that HOXC10 effectively alleviates pan-*KRAS*-mutant lung cancer with bone metastasis in the NOD1/ERK axis-dependent manner, and support further development of an effective combinatorial strategy for this kind of disease.

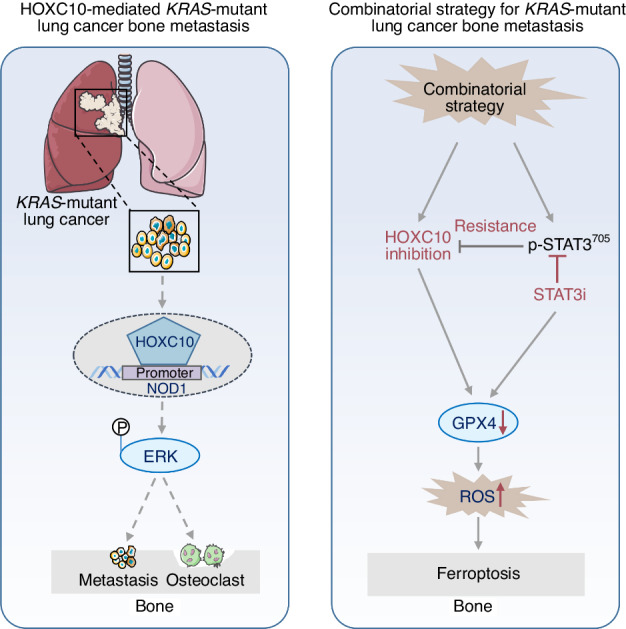

## Introduction

Lung cancer is one of the most common cancers and the leading cause of cancer-related deaths worldwide.^1^ Because the early stages of lung cancer are often without symptoms,^2^ it is usually diagnosed when the disease has progressed to local or systematic metastasis. Bone is a common site of metastasis in different primary cancers, and 30%-40% of lung cancer patients develop bone metastasis during the course of the disease.^3,4^ However, the genetic differences between primary lung cancer and matched bone metastasis are not yet fully understood. The mutation frequency of *KRAS* in lung cancer patients shows a metastatic site-dependent variation, and approximately 30% of all pan-*KRAS*-mutant lung cancers progress to bone metastasis.^5,6^ Mutation of *KRAS* is a predictive factor for treatment efficacy, as well as a prognostic factor for disease progression. Strikingly, a significant prognostic effect of KRAS status was found only in bone metastases by analyzing different subcohorts of lung cancer metastatic sites, including the lungs, bone, adrenal gland, brain, pleura and liver.^7^ The presence of *KRAS* mutation is related to markedly worse outcomes in bone metastatic cases.^7,8^ Currently, a few promising drugs targeting biomarkers in advanced metastatic lung cancer patients harboring various mutations are under investigation. Sotorasib, a selective KRAS inhibitor that specifically targets KRAS^G12C^ has been approved by the U.S. Food and Drug Administration for locally advanced or metastatic non-small-cell lung cancer harboring KRAS^G12C^ mutation.^9,10^ However, the major resistance mechanism for KRAS^G12C^ inhibitors was through RAS-ERK reactivation or a novel KRAS switch-II pocket mutation has emerged.^11,12^ The other strategy has been proposed to target Ras effectors, including MEK. However, MEK inhibitors demonstrate low efficacy as single agents or when combined with chemotherapy.^11^ More importantly, the most frequently mutated forms of KRAS, such as KRAS^G12D^ and KRAS^G12V^, are still undruggable.^10^ Therefore, the development of promising therapeutic strategies targeting pan-KRAS pathway effectors or pan-KRAS-associated proteins is urgently needed.

Homeobox C10 (HOXC10), a member of the homologous box superfamily, is closely associated with tumorigenesis^13^ and can be employed as a biomarker for cancer diagnosis or prognostic prediction. A recent study showed that upregulation of HOXC10 activates PI3K/AKT signal transduction to induce glioblastoma cell proliferation and lead to poor prognosis in glioblastoma.^14^ In breast cancer, suppressing the function of HOXC10 may be a potential strategy to overcome chemotherapeutic resistance.^15^ Metastasis is the main cause of low survival rates in cancer patients. Recent research has demonstrated that HOXC10 expression is significantly associated with tumor metastasis and invasion in different tumors. In gastric cancer, HOXC10 is highly expressed, and HOXC10 upregulation promotes gastric cancer cell proliferation and metastasis via modulation of NF-κB.^16,17^ In melanoma, HOXC10 promotes the growth and migration of melanoma by modulating Slug to activate YAP/TAZ signaling pathway.^18^ HOXC10 can activate the expression of MTFR2 to modulate the proliferation/invasion/migration of colorectal cancer cells.^19^ Moreover, previous studies suggest that HOXC10 is one of the most pivotal genes that reflects the degree of malignancy of lung cancer by RNA-seq analysis and HOXC10 downregulation significantly impairs tumor volumes, invasion and migration of lung cancer cells.^20,21^ Given that the function of HOXC10 as a pro-oncogene is required for malignant transformation and tumor metastasis, we speculate that HOXC10 may be linked in some way to the oncogenic response in pan-*KRAS*-mutant lung cancer bone metastasis. However, the biological function of HOXC10 in *KRAS*-mutant lung cancer bone metastasis is still unclear.

In the present study, we found that HOXC10 is a lynchpin among the most highly altered genes and promotes osteolytic bone metastasis in bone metastatic cells and clinical samples. Mechanistically, HOXC10 directly binds to the Nucleotide-binding oligomerisation domain (NOD)-containing protein 1(NOD1) promoter and regulates the NOD1/ERK signaling pathway. In addition, we assessed the therapeutic efficacy in vivo and found that *HOXC10* knockdown could inhibit bone metastasis until 21 days after intracardiac injection due to activation of JAK/STAT signal transduction, especially IL-6/JAK/STAT3 signaling pathway. Altogether, this study reveals a previously unrecognized role of HOXC10 in pan-*KRAS*-mutant lung cancer bone metastasis, and suggests HOXC10 as a therapeutic target for treating this intractable disease.

## Results

### HOXC10 expression is associated with pan-*KRAS*-mutant lung cancer bone metastasis

**Fig. 1:**
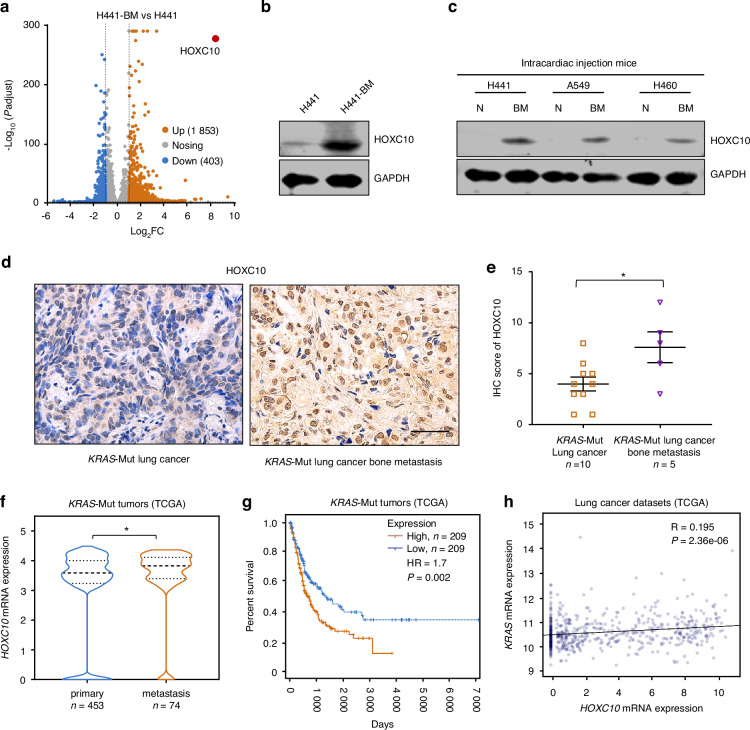
HOXC10 expression is associated with pan-*KRAS*-mutant lung cancer bone metastasis. **a** Volcano plot illustrating HOXC10 involved in differentially expressed genes. 678 down-regulated genes and 386 up-regulated genes in H441-BM vs. H441 cells. Fold change, >1.5; *P*adjust, <0.05. **b** The protein expression levels of HOXC10 in H441-BM (bone metastasis) vs. H441 cells. **c** The protein expression levels of HOXC10 in adjacent normal tissue and lung cancer bone metastasis tissue after mice intracardiac injection 6 weeks. **d** Representative IHC images for HOXC10 staining in *KRAS*-Mutant primary lung cancer and lung cancer bone metastasis samples. Scale bars, 50 μm. **e** The HOXC10 IHC staining for (*n* = 15), *KRAS*-Mut (*n* = 10) of lung cancer samples and *KRAS*-Mut (*n* = 5) of lung cancer bone metastasis samples were quantified. **f** HOXC10 expression levels in primary (*n* = 453) and metastasis (*n* = 74) tissues samples from *KRAS* mutation patients in the TCGA *KRAS* mutation Pan cancer dataset. **g** Kaplan-Meier survival curves for the indicated groups in the TCGA *KRAS* mutation Pan cancer dataset. HOXC10 high expression group (*n* = 209) displayed decreased survival compared to that of HOXC10 low expression group (*n* = 209). **h** Scatterplot showing the correlation between *KRAS* mRNA expression levels and *HOXC10* mRNA expression levels in the TCGA *KRAS* mutation lung cancer dataset. R, Pearson’s correlation co-efficient. Data in (**e**) shown as mean ± s.e.m. Panels (**e**, **f**) was performed unpaired two-sided Student’s *t* test, and (**g**) performed log-rank test, *P < 0.05

To explore the key genetic alterations in bone metastasis of *KRAS*-mutant lung cancer development, we applied RNA sequencing (RNA-seq) to compare metastatic lung cancer cell line H441-BM with primary lung cancer cell line H441. Bioinformatic analysis identified 1 853 upregulated genes and 403 downregulated genes in H441-BM compared to H441 cells (Fold change, >2.0; *P*adjust <0.05; Fig. 1a). Among the top 5 genes highly expressed in H441-BM cells from the RNA-seq, we selected HOXC10 in *KRAS*-mutant lung cancer bone metastasis for the reason that HOXC10 triggers metastasis in various malignancies, and it has been implicated as a pro-oncogene for malignant transformation.^13,18,20,21^ In agreement with the results of RNA-seq, the protein expression levels of HOXC10 were markedly increased in H441-BM cells (Fig. 1b).

To investigate the link between *KRAS*-mutant variants and HOXC10 expression, we obtained spine metastatic tissue with KRAS mutations including G12V/S and Q61H that mutation commonly occur in human lung cancer. Immunoblot analysis showed that the protein expression levels of HOXC10 in mouse lung cancer spine metastatic tissue were significantly higher than those in adjacent normal tissue (Fig. 1c), reinforcing the general role of HOXC10 in response to different KRAS mutant variants. We also explored the clinical significance of HOXC10 expression in different *KRAS*-mutant lung cancer patients with/without bone metastasis. Immunohistochemical results demonstrated a much higher protein expression level of HOXC10 in the bone metastasis subgroups than in the primary subtype (Fig. 1d, e).

Next, we analyzed the expression of HOXC10 in the primary and metastasis subgroups of pan-*KRAS*-mutant cancer using The Cancer Genome Atlas (TCGA) datasets. The results showed that HOXC10 mRNA levels were dramatically higher in the metastasis subgroups than in the primary subtype (Fig. 1f). We further explored the association between HOXC10 expression and the survival outcomes of patients in *KRAS*-mutant lung adenocarcinoma (LUAD) subgroups using TCGA datasets. It was found that the patients with high HOXC10 expression had a worse survival rate than those with low HOXC10 expression (Fig. 1g). Analysis of human LUAD datasets derived from TCGA datasets revealed that *KRAS* expression level was positively correlated with *HOXC10* expression level (Fig. 1h). These data indicate that HOXC10 overexpression may represent an independent risk factor affecting bone metastasis and the prognosis of *KRAS*-mutant cancer patients.

### *HOXC10* inhibition impairs cell growth and metastatic capacity in *KRAS*-mutant lung cancer

**Fig. 2:**
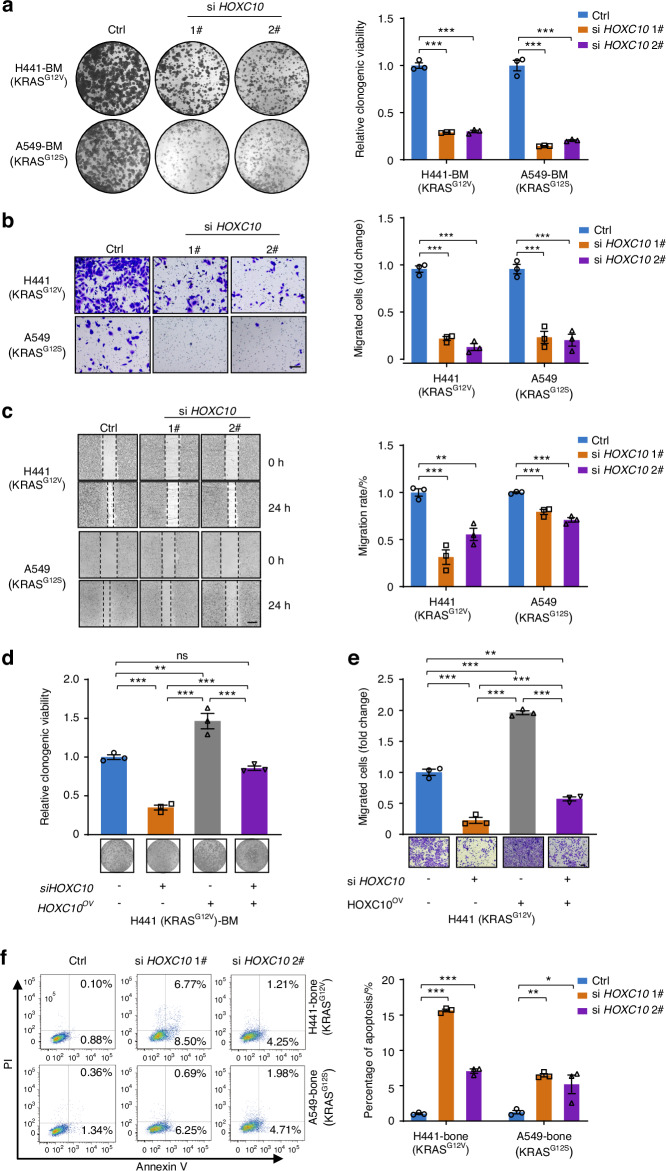
*HOXC10* inhibition impairs cell growth and metastatic capacity in *KRAS*-mutant lung cancer cells in vitro. **a** The colony-formation ability analysis of H441-BM and A549-BM cells transfected with the indicated siRNAs. The relative clonogenic viability was normalized to vehicle-treated control. **b** The migration ability of H441 and A549 cells transfected with the indicated siRNAs for 48 h were detected by transwell assays. The migrated cells were normalized to vehicle-treated control cells. Scale bars, 50 μm. **c** The migration ability of H441 and A549 cells transfected with the indicated siRNAs for 48 h were detected by was assessed by the wound healing assay. The migration rate was normalized to vehicle-treated control cells. Scale bars, 100 μm. **d**, **e** Ectopically expressed HOXC10 restored the clonogenic growth and migration ability of *HOXC10*-depleted cells. The relative clonogenic viability (**d**), and migration ability (**e**) analysis of indicated cells transfected with the indicated siRNAs. Scale bars, 50 μm. **f** Apoptosis of indicated cells were detected by flow cytometry. H441-bone and A549-bone cells were transfected with 20 nmol/L siRNAs targeting *HOXC10* for 48 h. The apoptosis cells were calculated by normalizing the untreated group as 100%. Data in (**a**–**f**) represent the mean ± s.e.m. of three technical replicates, representative of three independent experiments with similar results. Panels (**a**–**e**) were performed one-way ANOVA with Tukey’s multiple comparison test, *P < 0.05, **P < 0.01, ***P < 0.001

We next evaluated whether activated HOXC10 coordinated with oncogenic KRAS and played a promoting role in bone metastasis of lung cancer development. First, we assessed the effects of *HOXC10* knockdown on cancer cell growth in *KRAS*-mutant lung cancer bone metastasis cell lines. Our results showed that the cell growth inhibition rates of the *HOXC10* inhibition reached 53.2%–85.3% compared to those of the empty vector group of *KRAS*-mutant lung cancer bone metastasis cells, according to colony formation, CCK8, LDH release, and EDU assays (Fig. 2a and Fig. S1A–D). In addition, transwell assay (Fig. 2b) and wound healing assay (Fig. 2c) were conducted to assess the effect of *HOXC10* depletion on cell migration. Results from both experiments are consistent and collectively indicate migratory ability of lung cancer cells exhibited 20.6%–73.7% inhibition after *HOXC10* knockdown (Fig. 2b, c). The reduced growth (Fig. 2d) and migration (Fig. 2e) of H441 cells following *HOXC10* depletion could be rescued by HOXC10 overexpression, suggesting a HOXC10-dependent effect (Fig. 2d, e). We measured the apoptotic capacity by Annexin V staining and observed that the apoptosis of *KRAS*-mutant lung cancer bone metastasis cells increased 5.2-fold to 15.6-fold after HOXC10 knockdown (Fig. 2f). Meanwhile, the expression of BCL2 decreased and the expression of BAX increased after *HOXC10* depletion (Fig. S1E). Collectively, these findings demonstrate that *HOXC10* depletion can suppress proliferation and migration and promote apoptotic ability in *KRAS*-mutant lung cancer bone metastasis cells.

### *HOXC10* inhibition attenuates osteolytic bone metastasis

**Fig. 3:**
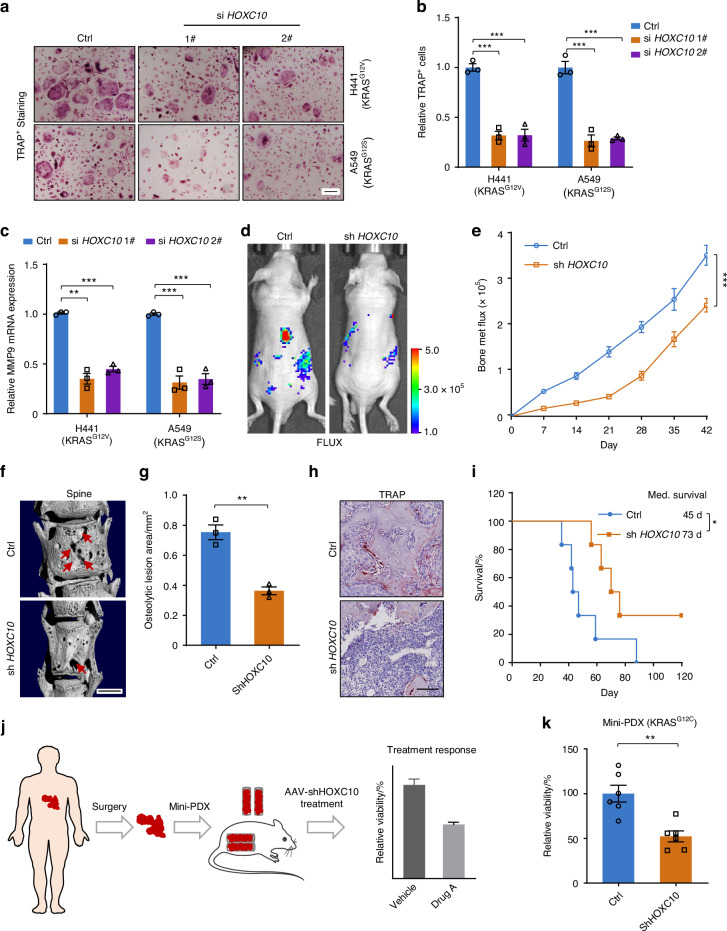
*HOXC10* inhibition attenuates osteolytic bone metastasis. **a**, **b** TRAP staining for primary preosteoclasts induced by H441 and A549 cells CM (**a**). The TRAP-positive multinucleated osteoclasts were analyzed (**b**). **c** qPCR validation of *MMP9* mRNA levels in H441 and A549 cells. **d** Representative IVIS bioluminescence imaging of H441 cells injected into BALB/c nude mice (6 weeks) via intracardiac injection at day 42 (*n* = 6 per group). **e** The growth of bone metastasis in (**d**) was quantified. **f** Representative micro-CT images of spine from the moribund intracardiac injection mice. Red arrows, osteolytic lesions. Scale bars, 1 mm. **g** Bar graph of quantitative osteolytic lesions of spine from the moribund mice (*n* = 3 per group). **h** Representative TRAP staining in bone of mice (*n* = 3 per group). **i** Survival curves for indicated mice (*n* = 6 per group). Scale, 50 μm. **j** Scheme of the mini-PDX models. **k** The relative cell viability shows a significant decrease in AAV-shHOXC10 mice post AAV treatment (*n* = 6 per group). Scale bars, 100 μm. Data in (**b**) represent three technical replicates, representative of three independent experiments with similar results. Data in (**b**, **c**, **e**, **g**, **k**) shown as mean ± s.e.m., (**b**) was performed one-way ANOVA with Tukey’s multiple comparison test, **c**, **g**, **k** performed unpaired two-sided Student’s *t* test, (**h**) performed log-rank test, *P < 0.05, **P < 0.01, ***P < 0.001

As the bone metastasis of lung cancer cells can lead to osteolytic lesions and induction of osteoclastogenesis,^22,23^ we next tested whether HOXC10 drives *KRAS*-mutant lung cancer cells to trigger osteoclastogenesis. Preinduced primary preosteoclasts were exposed to conditioned medium from H441 and A549 *KRAS*-mutant lung cancer cells with or without *HOXC10* knockdown. Tartrate-resistant acid phosphatase (TRAP) staining revealed that the inhibition rates TRAP^+^ osteoclasts induced by the conditioned medium from *HOXC10*-inhibited cancer cells reached 55.2%–65.2% compared to those of the TRAP^+^ osteoclasts induced by control cell conditioned medium (Fig. 3a, b). MMP9 is the biomarker of osteoclast differentiation.^24^ Furthermore, qPCR was performed and substantial 67.9%–76.3% reductions in MMP9 transcription was observed from *HOXC10* knockdown cancer cells conditioned medium induced osteoclasts (Fig. 3c). These results indicate that HOXC10 is involved in osteolytic bone metastasis and can promote osteolytic activity in the bone microenvironment.

Next, the conserved efficacy of *HOXC10* inhibition was also tested in an intracardiac injection mouse model in vivo. Our bioluminescence imaging results showed that loss of *HOXC10* significantly decreased bone metastasis in *KRAS*-mutant lung cancer (Fig. 3d, e and Fig. S2A). The major bone metastatic sites of spine were further confirmed by micro-CT imaging, which revealed significantly reduced bone destruction (Fig. 3f) and osteolytic bone lesions (Fig. 3g) in *HOXC10* knockdown group than in control group. The osteoclast number were decreased in *HOXC10* knockdown group (Fig. 3h). Moreover, Kaplan–Meier survival tests indicated that HOXC10 downregulation significantly prolonged metastasis-free survival in the target mice to a median of 73 days compared with the median of 45 days in control mice (Fig. 3i). To further explore the clinical benefit of HOXC10 inhibition in patients, we constructed a mini patient-derived xenograft (mini-PDX) model^25,26^ with KRAS^G12C^ mutant lung cancer bone metastasis (Fig. 3j). Effect of adeno-associated virus vector (AAV)-mediated HOXC10-knockdown treatment was evaluated in mini-PDX model. In vivo administration of AAV-shHOXC10 led to significant attenuation of tumor development (Fig. 3k).

### *HOXC10* inhibition impairs *KRAS*-mutant lung cancer bone metastasis by inactivating the NOD1/ERK axis

**Fig. 4:**
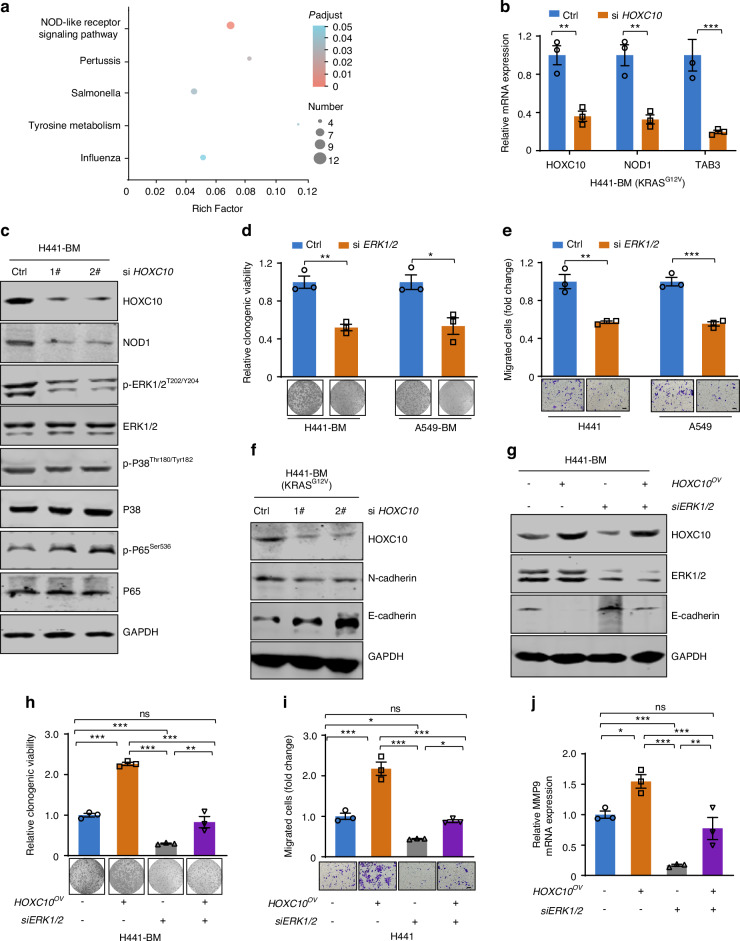
*HOXC10* inhibition impairs *KRAS*-mutant lung cancer bone metastasis by inactivating the NOD1/ERK axis. **a** The top 5 altered pathways of the RNA-seq data are shown (*n* = 3). **b** qPCR validation of representative differentially expressed genes by *HOXC10* loss in H441-BM cells. **c** *HOXC10* knockdown reduced NOD1 and p-ERK1/2^T202/Y204^ expression but not p-P38^Thr180/Tyr182^ and p-P65^Ser536^. Cells transfected with the indicated siRNAs for 48 h. The level of proteins was examined by western blot assay. **d** The colony-formation ability analysis of cells transfected with the indicated siRNAs. The relative clonogenic viability was normalized to vehicle-treated control. **e** The migration ability of transfected with the indicated siRNAs for 48 h were detected by transwell assays. The migrated cells were normalized to vehicle-treated control cells. Scale bars, 50 μm. **f** Cells transfected with the indicated siRNAs for 48 h. The level of E-cadherin and N-cadherin was examined by western blot assay. **g**–**j** The relative protein levels (**g**), colony-formation ability (**h**), migration ability (**i**), Scale bars, 50 μm, and *MMP9* mRNA levels (**j**) of indicated cells transfected with the indicated siRNAs. Data in (**b**, **d**, **e**, **h**, **i**, **j**) represent the mean ± s.e.m. of three technical replicates, representative of three independent experiments with similar results. Panels (**b**, **d**, **e**) were performed unpaired two-sided Student’s *t* test, (**h**, **i**, **j**) performed one-way ANOVA with Tukey’s multiple comparison test, *P < 0.05, **P < 0.01, ***P < 0.001, ns not significant

We next clarified the underlying mechanism by which *HOXC10* downregulation suppressed *KRAS*-mutant cancer growth and bone metastasis by utilizing genome-wide RNA-seq analysis. RNA-seq was conducted in *KRAS*-mutant lung cancer bone metastasis cells of H441-BM upon *HOXC10* knockdown. The enriched pathways of the differentially expressed genes between the *HOXC10*-knockdown and control groups were analyzed. The results demonstrated that the NOD-like receptor signaling pathway was enriched and was ranked highest among the altered pathways (Fig. 4a). These findings were further validated by real-time qPCR analysis of representative NOD-like receptor signaling pathway components, including *NOD1* and *TAB3* (Fig. 4b).

The NOD-like receptor signaling pathway is a subgroup of pattern recognition receptors that act as innate immune sensors of pathogen- and danger-associated molecular patterns.^27^ It has also been reported to be associated with many types of cancers.^28–30^ Several mechanisms underlying that NOD1 recognises bacterial peptidoglycan component diaminopimelic acid (DAP) to initiate an inflammatory response by interacting with receptor-interacting protein 2 kinase (RIP2) to trigger pathways downstream of MAPK/ERK, MAPK/P38, and NF-κB.^27^ To investigate whether the key downstream pathway of NOD1 was regulated by *HOXC10* inhibition, the MAPK/ERK, MAPK/P38, and NF-κB pathways were investigated by Western blot analyses. Our results showed that *HOXC10* knockdown decreased NOD1 and p-ERK^T202/Y204^ expression but not p-P38^Thr180/Tyr182^ and p-P65^Ser536^ expression in A549-BM and H441-BM cells at the protein level (Fig. 4c and Fig. S2B), indicating that HOXC10 only modulated the NOD1/ERK axis. Given that MAPK-ERK signal is the downstream of HOXC10, we measured the effects of ERK1/*2* knockdown on cell proliferation and migration and found that silencing *HOXC10* significantly suppress cells growth (Fig. 4d) and migration (Fig. 4e). This growth and migration inhibition activity was similarly to HOXC10 knockdown, as expected.

The acquisition of mesenchymal traits through the epithelial-mesenchymal transition (EMT) has been shown to promote bone metastatic properties,^31^ we determined whether EMT was regulated by HOXC10. Immunoblot analysis showed that ERK1/2 knockdown significantly decreased N-cadherin expression and increased E-cadherin expression (Fig. 4f and Fig. S2C). Reciprocally, ectopically expressed HOXC10 decreased E-cadherin expression, and inactivation of E-cadherin was blocked by simultaneous ERK1/2 knockdown (Fig. 4g), suggesting the MAPK-ERK signal is responsible for EMT process. On the other hand, when transfected in combination of ectopic expression of HOXC10 plasmids and siERK1/2, HOXC10 promoted growth (Fig. 4h and Fig. S2D) and migration (Fig. 4i and Fig. S2D), which were partially abrogated by ERK1/2 inhibition (Fig. 4i). Furthermore, qPCR results showed a marked increase in MMP9 transcription was observed from *HOXC10* overexpression cancer cells conditioned medium induced osteoclasts (Fig. 4j), which were partially abrogated by ERK1/2 inhibition (Fig. 4j). Taken together, it appears that HOXC10-mediated cell proliferation, migration, and osteolytic activity by activation of the NOD1/ERK signal.

### HOXC10 directly binds to *NOD1* promoter and promotes its expression

**Fig. 5:**
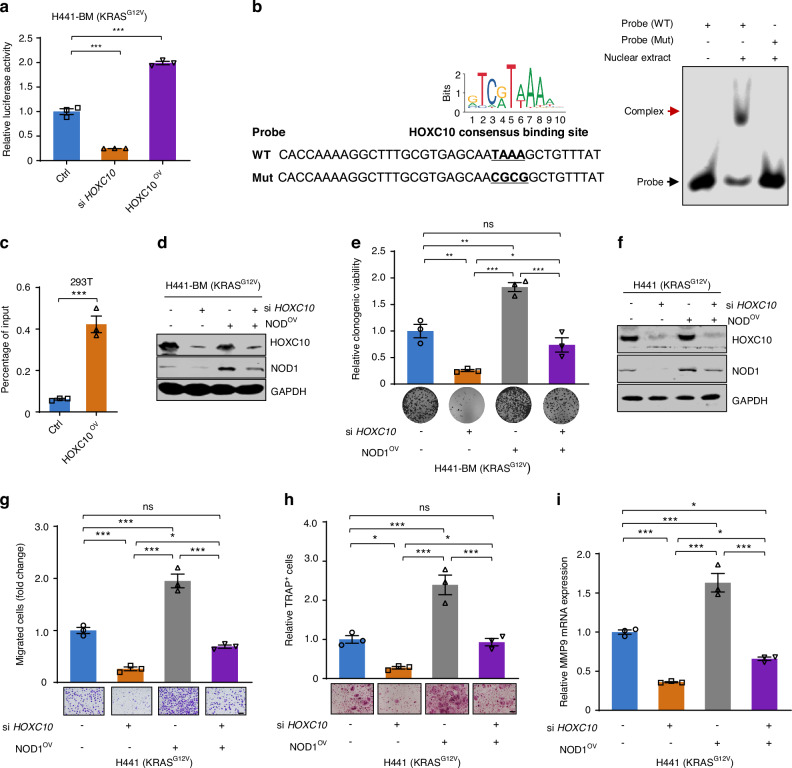
HOXC10 directly binds to NOD1 promoter and promotes its expression. **a** HOXC10 regulated the *NOD1* promoter activity. H441-BM cells were then harvested for a luciferase assay. **b** Electrophoretic mobility shift assay. The sequence of HOXC10 containing *NOD1* promoter binding site (*Left*) using the JASPAR database. The WT and Mut probes of NOD1 are shown (*left*). The mutated site is indicted in red. The binding complex of indicated probes and HOXC10 protein was indicated by arrows (*Right*). **c** ChIP assay showing the in vitro binding of HOXC10 and *NOD1* promoter. **d**, **e** Ectopically expressed NOD1 restored the clonogenic growth of *HOXC10*-depleted cells. The relative protein levels (**d**), and colony-formation ability (**e**) of indicated cells transfected with the indicated siRNAs. **f**–**i** The relative protein levels (**g**) Scale bars, 50 μm, migration ability (**i**), osteoclast differentiation ability (**h**) Scale bars, 100 μm, and *MMP9* mRNA levels (**j**) of indicated cells transfected with the indicated siRNAs. Data in (**a**, **e**, **g**, **h**, **i**) represent the mean ± s.e.m. of three technical replicates, representative of three independent experiments with similar results. Statistical analysis in (**a**, **e**, **g**, **h**, **i**) were performed one-way ANOVA with Tukey’s multiple comparison test, and in (**c**) performed unpaired two-sided Student’s *t* test, *P < 0.05, **P < 0.01, ***P < 0.001, ns not significant

Considering the positive correlation between HOXC10 and NOD1/ERK signal, we next sought to define the detail regulatory mechanism of *NOD1* gene expression by HOXC10. A *NOD1* reporter assay was conducted to examine the interaction between HOXC10 and NOD1. Luciferase reporter assay indicated that the activity of *NOD1* promoter was inhibited to 25.1% of its original level by *HOXC10* genetic silencing in H441-BM cells (Fig. 5a). In contrast, the activity of *NOD1* promoter increased to 2.0-fold of its original level by HOXC10 ectopical expression (Fig. 5a). Analysis of the human NOD1 promoter region using the JASPAR transcription factor database (http://jaspar.genereg.net/) predicted potential HOXC10 recognition site (Fig. 5b) responsible for transcriptional repression. Thus, electrophoretic mobility shift assays were performed, in which a 56-bp probe for *NOD1* promoter containing an HOXC10-binding site was exposed to purified HOXC10 protein. Noticeably, the DNA-protein complex-shifted band was obviously detected in the presence of the wild-type NOD1 probe (Fig. 5b). Conversely, NOD1 mutation in TAAA motif substantially reduced the NOD1 DNA binding activity of the HOXC10 complex (Fig. 5b), suggesting the specificity of HOXC10 binding to the NOD1 probe in TAAA motif. Next, we performed ChIP–qPCR analysis and the results further confirmed the presence of HOXC10 on the *NOD1* promoter in 293 T cells (Fig. 5c).

Moreover, ectopically expressed NOD1 in *HOXC10*-depleted cells apparently restored the colony-forming capability (Fig. 5d, e) and migration capacity in H441 cells (Fig. 5f, g). The number of TRAP^+^ osteoclasts induced by the conditioned medium from *HOXC10*-inhibited cancer cells were dramatically reduced and the reduced reversed by NOD1 overexpression (Fig. 5h) Furthermore, qPCR results showed a marked decrease in MMP9 transcription was observed from *HOXC10* knockdown cancer cells conditioned medium induced osteoclasts (Fig. 5i), which were partially abrogated by NOD1 overexpression (Fig. 5i), indicating a NOD1-dependent effect. Taken together, our results indicate that HOXC10 directly binds to the NOD1 promoter, which is functionally important for HOXC10 activation.

### STAT3 inhibition synergizes with *HOXC10* inhibition in vitro and in vivo

**Fig. 6:**
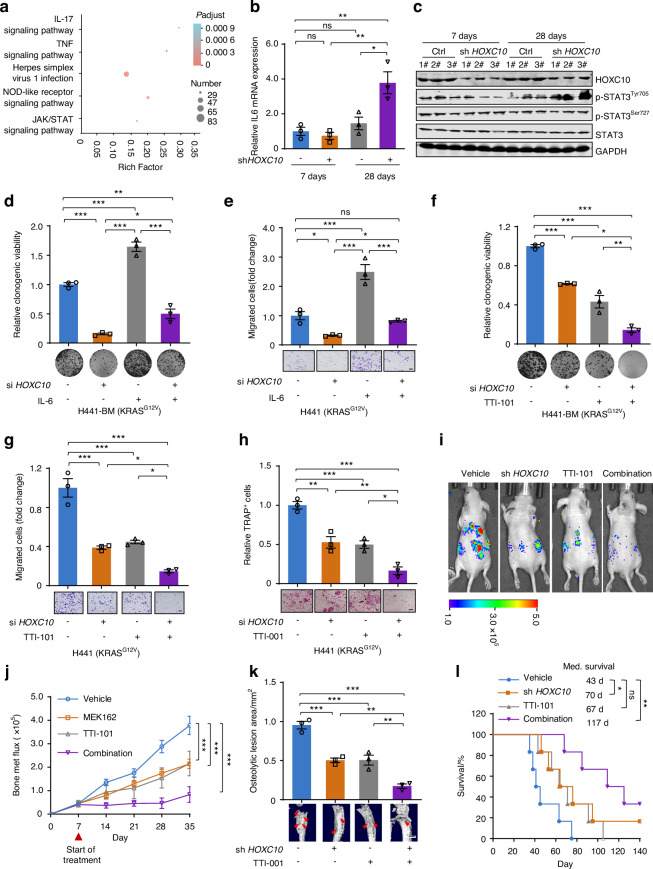
STAT3 inhibitor synergize with *HOXC10* inhibition in vitro and in vivo. **a** The top 5 altered pathways of the RNA-seq data. The RNA samples extracted from *HOXC10* knockdown bone metastasis tissues after intracardiac injection at 7 and 28 days (*n* = 3). **b** qPCR validation of *IL6* mRNA level. **c** Protein expression levels. Bone metastasis tumors were harvested at day 7, and day 28 in Fig. 3d. Two biologically independent samples per group from five independent samples are shown. **d**, **e** IL-6 treatment reduced *HOXC10* knockdown-mediated cytotoxicity. Cells were treated with IL-6 at the concentration of 20 ng/mL. The relative clonogenic viability (**d**) and migration ability (**e**) were normalized to vehicle-treated control. Scale bars, 50 μm. **f**, **g** TTI-101 treatment increased *HOXC10* knockdown-mediated cytotoxicity. Cells were treated with TTI-101 at the concentration of 4 μmol/L. The relative clonogenic viability (**f**) and migration ability (**g**) were normalized to vehicle-treated control. Scale bars, 50 μm. **h** The osteoclast differentiation ability of HOXC10 inhibition plus TTI-001 (4 μmol/L) was assessed. Scale bars, 100 μm. **i** Representative IVIS bioluminescence imaging of indicated mice at day 35. After 7 days intracardiac injection, mice were treated with TTI-101 (25 mg/kg) for an additional 28 days (*n* = 6 per group). **j** The growth of bone metastasis in (**i**) was quantified (*n* = 6 per group). **k** Representative micro-CT images of spine. Red arrows, osteolytic lesions. Bar graph of quantitative micro-CT analysis of osteolytic lesions of spine from the moribund mice (*n* = 3 per group). Scale bars, 1 mm. **l** Survival curves for indicated mice (*n* = 6 per group). Data in (**b**, **d**, **e**, **f**, **g**, **h**) represent three technical replicates, representative of three independent experiments with similar results. Data in (**b**, **d**, **e**, **f**, **g**, **h**, **j**) shown as mean ± s.e.m. Panels (**b**, **d**, **e**, **f**, **g**, **h**, **k**) were performed one-way ANOVA with Tukey’s multiple comparison test, (**j**) performed two-way ANOVA with Sidak’s multiple comparisons test, and (**l**) performed log-rank test, *P < 0.05, **P < 0.01, ***P < 0.001, ns not significant

Although *HOXC10* inhibition showed promising benefits in *KRAS*-mutant lung cancer bone metastasis in vivo (Fig. 3d, e), we found that the ability of inhibiting bone metastasis by *HOXC10* knockdown was significantly diminished 28 days after intracardiac injection (Fig. 3e). We hypothesize that this may be due to the development of treatment resistance to *HOXC10* inhibition 28 days after intracardiac injection. To understand the mechanism in *HOXC10* inhibition treatment resistance, the RNA samples extracted from *HOXC10* knockdown bone metastasis tissues after intracardiac injection at 7 and 28 days were subjected to RNA-seq analysis. The enriched pathways of the differentially expressed genes were further analyzed. Our results demonstrated that IL-17, TNF and JAK-STAT signaling pathways were enriched and were in the leading rank of altered pathways (Fig. 6a). It is well established that activation of JAK/STAT signaling pathway, especially IL-6/JAK/STAT3 signaling pathway, drives tumor metastasis^32,33^ and promotes drug resistance in lung cancer.^34–36^ Thus, we speculate that IL-6/JAK/STAT3 signaling pathway may confer resistance to HOXC10 knockdown in *KRAS*-mutant lung cancer bone metastasis. To infer the role of IL6/JAK/STAT3 signaling pathway in resistance to HOXC10 inhibition, qPCR was performed in bone metastasis tissue and a marked increase in IL-6 transcription was observed after intracardiac injection at 28 days (Fig. 6b). Next, we profiled the protein expression of p-STAT3^Tyr705^, p-STAT3^Ser727^ and STAT3 by Western blot analysis. *KRAS*-mutant lung cancer bone metastasis tissue exhibited higher p-STAT3^Tyr705^ levels at day 28 after intracardiac injection than at day 7 (Fig. 6c), suggesting that activation of IL6/JAK/STAT3 pathway may be involved in the enhanced resistance to *HOXC10* inhibition. Then, H441-BM cells were transfected with si*HOXC10* and then stimulated with IL-6, to activate IL6/JAK/STAT3 pathway. Notably, IL-6 treatment was able to stimulate p-STAT3^Tyr705^ expression in H441-BM cells (Fig. S3A). Furthermore, the phenomenon of IL6/JAK/STAT3 pathway mediated *HOXC10* inhibition resistance was further verified by colony formation and transwell assays. We found that the growth retardation of *HOXC10* depletion on H441-BM cells (Fig. 6d) and the antimetastatic effects of *HOXC10* depletion on H441 cells (Fig. 6e) were markedly decreased with IL-6 treatment. Based on these observations, TTI-101, a developed STAT3 inhibitor (STAT3i),^37^ along with *HOXC10* depletion, was further applied to examine the antigrowth and antimetastatic effects of the combinations on H441-BM cells (Fig. 6f, g). Our results showed that STAT3i effectively sensitized *HOXC10* depletion. Tartrate-resistant acid phosphatase (TRAP) staining revealed that the number and size of TRAP^+^ osteoclasts induced by the conditioned medium from STAT3 inhibition, together with *HOXC10*-inhibited cancer cells, were significantly deceased (Fig. 6h).

Next, we investigated whether STAT3i could sensitize cancer cells to *HOXC10* inhibition in vivo. Therefore, we evaluated the effects of TTI-101 on *HOXC10* inhibition sensitivity in intracardiac injection models. As expected, TTI-101 potentiated the inhibition efficacy of *HOXC10* knockdown on lung cancer metastatic capacity by bioluminescence imaging analysis (Fig. 6i, j). The major bone metastatic sites of spine were further confirmed by micro-CT imaging, which revealed significantly reduced bone destruction and osteolytic bone lesions (Fig. 6k) by dual *HOXC10* and STAT3 inhibition. We also investigated the survival benefit of HOXC10 inhibition plus STAT3i. Compared with the control group, *HOXC10* inhibition monotherapy had a limited effect on the survival of mice with intracardiac injection, whereas the addition of TTI-101 markedly prolonged survival, with an added median survival benefit of 47 days (Fig. 6l). Collectively, these results indicate that IL6/JAK/STAT3 pathway plays an important role in conferring *HOXC10* inhibition resistance.

### HOXC10 and STAT3 co-inhibition induces ferroptosis

**Fig. 7:**
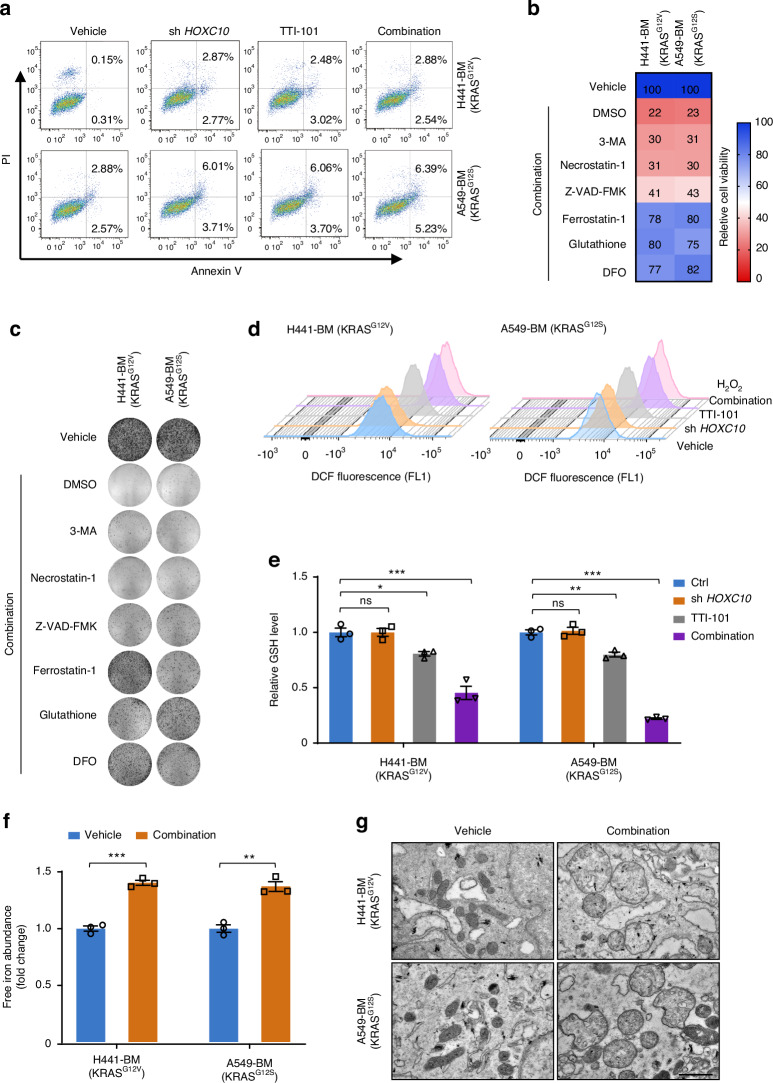
HOXC10 and STAT3 co-inhibition robustly triggers ferroptosis. **a** The apoptosis analysis of indicated cells treated with TTI-101 (4 μmol/L) for 72 h. The apoptosis cells were normalized to vehicle-treated control. Combination refers to HOXC10 inhibition plus TTI-101 cotreatment. **b**, **c** Cell death induced by HOXC10 inhibition plus TTI-101 cotreatment was rescued by ferroptosis inhibitors. Cells with or without knockdown of *HOXC10* were pretreated with HOXC10 TTI-101 for 12 h and then treated the indicated cell death inhibitors (3-MA-1 mmol/L, Necrostatin-1-20 μmol/L, Ferrostatin-1-2 μmol/L, Z-VAD-FMK-10 μmol/L, Glutathione-1 mmol/L, Deferoxamine (DFO-100 μmol/L)) for an additional 72 h. Effect on ferroptosis inhibitors rescuing cell death was assessed by cell viability assays (**b**) and clonogenic assays (**c**). **d** HOXC10 inhibition and TTI-101 (4 μmol/L) combination increased ROS production. Cells were treated with pharmacological or genetical approaches for 72 h. **e** HOXC10 inhibition and TTI-101 combination inhibited GSH level. Cells were treated with TTI-101 (4 μmol/L) or genetical approaches for 72 h. Relative GSH levels were normalized to vehicle-treated control. **f** HOXC10 inhibition and TTI-101 combination inhibited the intracellular concentrations of Fe^2+^. Cells were treated with TTI-101 (4 μmol/L) or genetical approaches for 72 h. Relative intracellular concentrations of Fe^2+^ was normalized to vehicle-treated control. **g** HOXC10 inhibition and TTI-101 combination reduced the mitochondrial cristae. Cells were treated with TTI-101 (4 μmol/L) or genetical approaches for 72 h. Relative intracellular concentrations of Fe^2+^ was normalized to vehicle-treated control. Representative Transmission electron microscopy (TEM) imaging of mitochondrial cristae. Scale bars, 1 μmol/L. Data in (**a**–**f**) represent three technical replicates, representative of three independent experiments with similar results.Data in (**e**, **f**) shown as mean ± s.e.m., (**e**) was performed performed one-way ANOVA with Tukey’s multiple comparison test, and (**f**) performed unpaired two-sided Student’s *t* test, *P < 0.05, **P < 0.01, ***P < 0.001, ns not significant

In addition, the mechanisms underlying the potency of STAT3 inhibition synergizes with HOXC10 inhibition were examined. Annexin V staining was conducted, and it was found that HOXC10 inhibition combined with TTI-101 treatment could not promote apoptotic cell death compared with either *HOXC10* inhibition monotherapy or TTI-101 death inhibitor 3-methyladenine (3MA), as well as zVAD-FMK and Necrostatin-1 that suppress apoptosis and necrosis^38^ (Fig. S4A), respectively, only marginally rescued cell death in response to HOXC10 and STAT3 co-inhibition treatment but effectively rescued cell death in response to their cognate pathway-specific inducers (Fig. 7a and Fig. S4B). On the contrary, the antioxidant ferrostatin-1, glutathione and deferoxamine, which are known to suppress ferroptosis,^38^ could effectively rescue cell death induced by HOXC10 and STAT3 co-inhibition, as evaluated by cell viability assays (Fig. 7b) and colony formation assays (Fig. 7c and Fig. S4C) in A549-BM and H441-BM cells. These results suggest that HOXC10 inhibition combined with TTI-101 treatment induces ferroptosis. Ferroptosis is a unique cell death pathway driven by iron-dependent lipid peroxidation.^39^ We examined the effect of HOXC10 inhibition combined with TTI-101 treatment on reactive oxidative species (ROS) levels using DCFH-DA flow cytometry measurements and observed a significant increase in ROS level (Fig. 7d) in A549-BM and H441-BM cells. Ferroptosis pathway can be induced by multiple mechanisms that regulate polyunsaturated fatty acids (PUFAs), labile iron levels, intracellular glutathione (GSH) pool, and the activity of GSH peroxidase 4 (GPX4), a selenoprotein that decreases phospholipid hydroperoxides through GSH and protects against ferroptotic cell death.^39,40^ Concomitantly, the cellular GSH (Fig. 7e) was found to be decreased. Next, the lipid ROS, and intracellular Fe^2+^ levels (biomarkers of ferroptosis) were detected. The results suggested that HOXC10 inhibition combined with TTI-101 treatment markedly increased the lipid ROS (Fig. S5A) and the intracellular concentrations of Fe^2+^ (Fig. 7f). Transmission electron microscopy (TEM) showed that HOXC10 inhibition combined with TTI-101 treatment reduced the mitochondrial cristae (Fig. 7g). We also detected the expression of ferroptosis-related proteins by Western blot. The results indicated that HOXC10 inhibition combined with TTI-101 treatment reduced the expression of GPX4 (Fig. S5B). In addition, we observed a marked decrease in GPX4 gene expression and an increase in ACSL4 expression in intracardiac injection mouse model after treatment (Fig. S5C). Altogether, these results demonstrate that HOXC10 inhibition and TTI-101 combination has dramatic effects on key ferroptosis mediators in *KRAS*-mutant lung cancer bone metastasis cells, including cellular ROS and GSH levels.

### STAT3i sensitizes *KRAS*-mutant lung cancer bone metastasis to MEK inhibitors

**Fig. 8:**
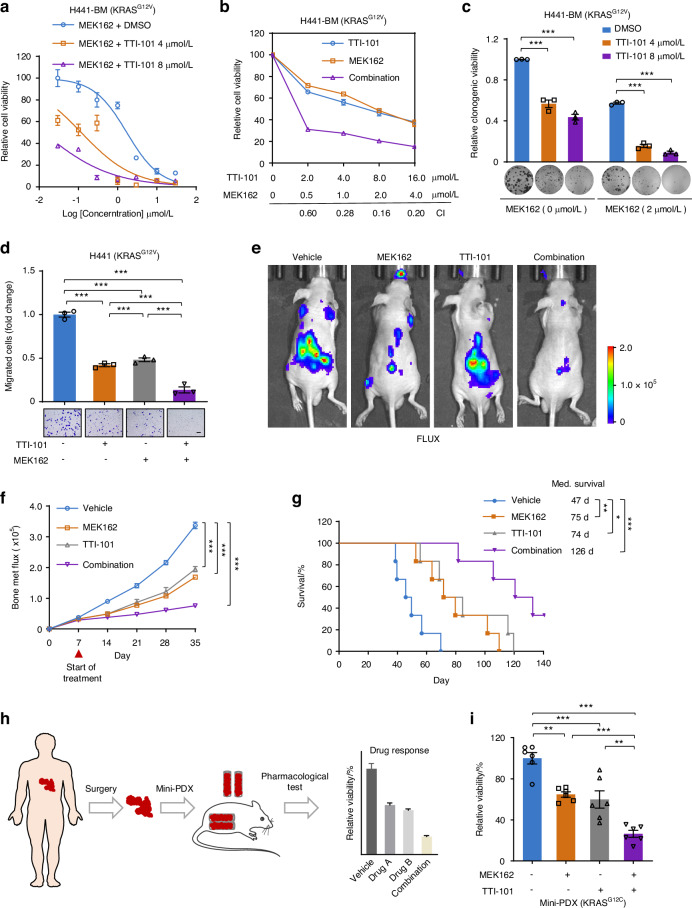
STAT3i sensitizes *KRAS*-mutant lung cancer bone metastasis to MEK inhibitor. **a** TTI-101 enhanced the inhibition efficacy of MEK162 in H441-BM cells. Combination refers to MEK162 (2 μmol/L) plus TTI-101 (4 μmol/L) cotreatment. Cells were treated with various concentrations of indicated inhibitors for 72 h. **b** Synergistic interaction between MEK162 (2 μmol/L) and TTI-101 (4 μmol/L) in H441-BM cells. Cells were treated with various concentrations of indicated inhibitors for 72 h. **c**, **d** TTI-101 (4 μmol/L) treatment increased MEK162 (2 μmol/L) mediated cytotoxicity. Cells treated with indicated inhibitors. The relative clonogenic viability (**c**) and migration ability (**d**) was normalized to vehicle-treated control. Scale bars, 50 μm. **e** Representative IVIS bioluminescence imaging of H441 cells injected into BALB/c nude mice (6 weeks) via intracardiac injection at day 35. After 7 days intracardiac injection, mice were treated with vehicle, TTI-101 (25 mg/kg), MEK162 (25 mg/kg) or combination for an additional 28 days (*n* = 6 per group). **f** The growth of bone metastasis in (**e**) was quantified. **g** Survival curves for indicated mice (*n* = 6 per group). **h** Scheme of the mini-PDX models. **i** The relative cell viability of mini-PDX treated with TTI-101 (25 mg/kg), MEK162 (25 mg/kg) or combination treatment for 7 days, and normalized to vehicle treatment (*n* = 6 per group). Data in (**c**, **d**) represent three technical replicates, representative of three independent experiments with similar results. Data in (**a**, **b**, **c**, **d**, **f**) shown as mean ± s.e.m. Panels (**c**, **d**, **i**) were performed one-way ANOVA with Tukey’s multiple comparison test, (**f**) performed two-way ANOVA with Sidak’s multiple comparisons test, and (**g**) using log-rank test, *P < 0.05, **P < 0.01, ***P < 0.001

Considering that MEK inhibitors (MEKis),^41^ key inhibitors of inactive p-ERK, have greater clinical transformation ability than current HOXC10 inhibitors, we further determined whether the clinically used drugs MEK162 and TTI-101 can sensitize to MEKi and STAT3i, respectively, both in vitro and in vivo. First, we applied different concentrations of MEK162 along with TTI-101 and assessed the antigrowth effects of different drug combinations in H441-BM cells. It was found that the STAT3 inhibitor remarkably improved the antigrowth effect of MEK inhibitor on H441-BM cells (Fig. 8a), and the CI values were all below 0.6 (Fig. 8b), indicating a synergistic effect. Next, the synergistic effect of MEKi and STAT3i was measured by a long-term colony formation assay and transwell assay. We found that TTI-101 inactivated STAT3 and significantly sensitized H441-BM cells to MEK162 with regard to clonogenic growth (Fig. 8c) and migration (Fig. 8d). Moreover, we used an intracardiac injection mouse model to further assessed the therapeutic efficacy of MEK162 plus TTI-101 combination therapy. Our results showed that treatment with the drug pair significantly prevented tumor metastasis in mice (Fig. 8e, f), as revealed by bioluminescence imaging. More encouragingly, MEK162 plus TTI-101 combination therapy produced a higher long-term survival advantage in mice (Fig. 8G). The biosafety of drug pair treatment was next investigated. Drug combinations showed a provocative effect with higher toxicity to lung cancer bone metastasis cells than that to normal than normal lung epithelial cells (Fig. S6A). Intriguingly, drug pair treatment did not significantly affect the weight of mice (Fig. S7A). Serum from the mice was used to evaluate liver and kidney function, and no significant functional abnormalities were found in the mice (Fig. S7B–D). The results of H&E staining in important organs showed that drug pair treatment had no significant systemic toxicity (Fig. S7E). The pharmacokinetic features of MEK162 and TTI-101 were then tested in BALB/c nude mouse. To this end, four nude mouse were administered MEK162 (25 mg/kg), and another four nude mouse were inoculated with TTI-101 (25 mg/kg) by intraperitoneal injection (i.p.). The mean plasma concentration-time profiles are shown in Fig. S7F–G. The main pharmacokinetic parameters in plasma, tumor and different tissues are summarized in Table S3–S6. The results indicated that in the four nude mouse that received an i.p. injection of 25 mg/kg MEK162, the plasma MEK162 level reached a peak concentration of 10 313 ± 6 291 ng/mL within 0.375 h with a t_1/2_ of 4.36 h. In the four nude mouse that received an i.p. injection of 25 mg/kg TTI-101, the plasma TTI-101 level reached a peak concentration of 2 057 ± 1 355 ng/mL within 0.875 h with a t_1/2_ of 3.64 h. These results demonstrated that MEK162 and TTI-101 shows bioavailability following intraperitoneal injection (i.p.), enabling in vivo efficacy.

To further explore the potential clinical application of drug combinations, we used the mini-PDX model^25,26^ with KRAS^G12C^ mutant lung cancer bone metastasis (Fig. 8h, i). *KRAS*-mutant lung cancer bone metastasis tumors showed higher sensitivity to cotreatment with MEK162 and TTI-101 than monotherapy. These results support the notion that synergistic MEK and STAT3 inhibition is an effective therapeutic strategy for *KRAS*-mutant lung cancer bone metastasis.

## Discussion

Lung cancer patients face the challenge of bone metastasis, which leads to poor survival. Accounts for 30% of lung cancer patients with bone metastasis harbor activating *KRAS* mutations.^7,8^ *KRAS* mutation is the primary cause of the markedly worse outcomes in lung cancer bone metastasis cases. While KRAS^G12C^ inhibitor has been approved for the treatment of advanced metastatic lung cancer,^42^ there is still a lack of effective therapeutic strategies for *KRAS*-mutant cases. Herein, we explored the association between HOXC10 expression and *KRAS*-mutant lung cancer bone metastasis. To our knowledge, this study is the first to identify HOXC10 as a lynchpin in pan-*KRAS*-mutant lung cancer bone metastasis. Interestingly, HOXC10 interacts with the NOD1 promoter to drive MAPK/ERK pathway as a de novo regulatory mechanism. Our experiments showed that *HOXC10* inhibition impeded the proliferation and migration of KRAS-mutant lung cancer cells in vitro and attenuated osteolytic bone metastasis in vivo. Intriguingly, inhibition of HOXC10 plus STAT3 inhibitor in combination was effective against *KRAS*-mutant lung cancer bone metastasis by triggering ferroptosis. These findings demonstrate how specific *HOXC10* knockdown confers therapeutic vulnerabilities, and suggest that combination treatment can lead to the inhibition of pan-*KRAS*-mutant lung cancer bone metastasis.

HOXC10 encodes a conserved DNA-binding homeodomain-containing transcription factor, which binds to the origins of replication for the assembly of replicative complexes.^43^ Abnormal HOXC10 expression has been reported in a variety of cancers. Dysregulation of HOXC10 is markedly associated with tumor invasion and metastasis.^20,25,44^ HOXC10 promotes tumor metastasis via the EMT signaling pathway in ovarian cancer^45^ and oral squamous cell carcinoma.^46^ Bone is a common site of metastasis for lung cancer. Primary tumors and the circulating factors derived from them can condition target cells residing in the bone to support metastatic cell colonization. EMT and matrix metalloproteinases (MMPs) are important for driving metastatic cell to colonize in bone. These findings support HOXC10 expression as a lynchpin for lung cancer cells of bone colonization. HOXC10 accelerates cancer progression by activating both MAPK^47^ and PI3K/AKT pathways,^48^ which are the main downstream pathways of KRAS.^49^ Study have shown that BET bromodomain epigenetic signaling interferes with the bone-associated tumor vicious cycle.^50^ More importantly, *HOXC10* expression inversely correlated with PRC2- BET bromodomain epigenetic signaling activity.^51^ These findings support a link between HOXC10 dysregulation and *KRAS*-mutant lung cancer with bone metastasis. One study shows that HOXC10 is one of the most pivotal genes that reflects the degree of malignancy of lung cancer, and downregulation of HOXC10 significantly decreases lung cancer metastasis,^20,21^ which are consistent with our results. We found that HOXC10 may serve as a novel biomarker of *KRAS*-mutant lung cancer bone metastasis, implying the multifaceted role of HOXC10 in *KRAS*-mutant lung cancer bone metastasis and broadening the application of HOXC10-based therapy.

HOXC10 affects tumorigenesis by modulating cell proliferation and metastasis. The tumor regulatory network of HOXC10 is complex. A reported study showed that HOXC10 promotes gastric cancer cell migration and invasion and enhances the activity of NF-κB pathway.^52^ HOXC10 also upregulates the phosphorylation levels of extracellular signal-regulated kinase ERK, c-Jun N-terminal kinase (JNK) and p38.^16^ The NF-κB, ERK and p38 pathways are also the main downstream pathways of NOD1.^28^ The working patterns of NOD1 and downstream pathways are similar to those of HOXC10 and downstream pathways. In addition, the acquisition of stimulation of NOD1 has been shown to promote potentiates liver metastasis of colorectal cancer.^53^ In our study, we found that HOXC10 regulated *KRAS*-mutant lung cancer bone metastasis via activation of the NOD1/ERK axis (Fig. 4). Our HOXC10 /NOD1/ERK axis pathway fits with NOD1 regulating of cancer metastasis. Therefore, HOXC10 occupancy of the *NOD1* promoter was required for NOD1/ERK pathway activation and its biological functions in regulating cell survival and metastasis (Fig. 4), implying a novel regulatory mechanism. These observations provide novel insights into *KRAS*-mutant lung cancer bone metastasis by mechanistically linking HOXC10 and the NOD1/ERK axis.

IL6/STAT3 pathway is activated in *KRAS*-driven tumors and can contribute to tumor occurrence^54,55^ and drug resistance^34–36^ in lung cancer. In this study, we found that IL6/STAT3 pathway confers resistance to HOXC10 inhibition in *KRAS*-mutant lung cancer bone metastasis and that activation of p-STAT3^Tyr705^ further promotes cell survival after HOXC10 inhibition (Fig. 5). IL-6 treatment stimulates p-STAT3Tyr705 expression and HOXC10 depletion could not change its expression (Fig. S3), suggesting a HOXC10-independent effect. We propose a regimen of dual inhibition of HOXC10 and STAT3 for improving the effectiveness of HOXC10 inhibition alone in *KRAS*-mutant lung cancer bone metastasis. More importantly, we found that combining HOXC10 inhibition and STAT3i could confer a marked survival benefit in this aggressive, refractory tumor in an intracardiac injection mouse model (Fig. 5). HOXC10 inhibition plus STAT3 inhibition could induce ferroptotic cell death. It has been previously shown that STAT3 is a positive regulator of ferroptosis in *KRAS*-mutant pancreatic ductal adenocarcinoma cell lines.^56^ Strikingly, we found an increase in intracellular GSH and ROS levels in response to HOXC10 inhibition plus STAT3 inhibition in *KRAS*-mutant lung cancer bone metastasis cell lines (Fig. 6), implying that this drug combination, not only is an effective cancer therapy, but also can be employed as an experimental tool to clarify the underlying mechanism of ferroptosis, especially in *KRAS*-mutant lung cancer bone metastasis. Nevertheless, more detailed experiments are needed to further clarify the interaction mechanisms of HOXC10 and p-STAT3^Tyr705^ in conferring HOXC10 inhibition resistance and triggering ferroptosis. Besides, it is important to evaluate the benefit of STAT3i as a combination regimen to overcome HOXC10 inhibition-mediated resistance in the near future.

Besides, our findings showed that the NOD1/ERK axis exerted its biological function via HOXC10 activation. Considering that MEK inhibitor (MEKi), a key inhibitor of inactive p-ERK, is more translatable to the clinic than the current HOXC10 inhibitors, a feasible combination of MEKi/STAT3i is proposed to circumvent resistance. Notably, a regimen of dual inhibition of HOXC10 and STAT3 is established to improve the effectiveness of HOXC10 inhibition alone in *KRAS*-mutant lung cancer bone metastasis. More importantly, we found that combining HOXC10 inhibition and STAT3i could confer a marked survival benefit in this aggressive, refractory tumor in an intracardiac injection mouse model (Fig. 7). The crucial role of STAT3 pathway in MEKi resistance is also supported by a recent study demonstrating the synergistic effect of STAT3i and MEKi in *KRAS*-mutant lung cancer.^57^

Taken together, our work indicates that HOXC10/NOD1/ERK signaling can be a promising target for preventing and treating lung cancer bone metastasis. This will help inform the combination use of FDA-approved MEK inhibitors and STAT3 inhibitor in an ongoing phase I clinical trials (ClinicalTrials.gov ID:NCT03195699) to treat with *KRAS*-mutant lung cancer bone metastasis patients.

## Materials and methods

### Reagents

Apoptosis inducer 7, Z-VAD-FMK, RIP1/RIP3/MLKL activator 1, Necrostatin-1, Rapamycin, 3-Methyladenine, Ferrostatin-1, Glutathione, Deferoxamine, TTI-101 and MEK162 were obtained from MedChemExpress (Monmouth Junction, New Jersey, USA). All compounds were formulated in 100% dimethyl sulfoxide (DMSO) and aliquoted for long term storage at -20 °C.

### Cell Lines

H441, H838, and A549 cells were obtained from ATCC (Manassas, VA). H838-KRAS^G12D^ cells were generated by importing the KRAS(G12D) variant (Addgene, Cambridge, MA, USA) into H838 cells. A549-BM, H441-BM, H838-bone, and H838-bone KRAS^G12D^ cells underwent in vivo selection by intracardiac injection model, leading to spine metastatic cells. Cell lines were cultured in RMPI-1640 medium supplemented with 10% fetal bovin serum (FBS).

### Patient samples

Mutation status of *KRAS* in primary (*n* = 25) and bone metastasis (*n* = 21) human lung cancer tissues (from Fudan University Shanghai Cancer Center) were determined by Sanger sequencing. FFPE sections were stained with HOXC10 antibody from Abcam (ab153904, 1:100 dilution, Cambridge, MA). The images of IHC (stained with HOXC10 antibody) slides were obtained using a whole-slide scanner (ScanScope AT; Aperio). Staining was quantified as previously described.^58,59^

### Western blotting analysis

Western blotting assays were performed as previously described.^58^ Briefly, equal amounts (40-80 μg) of cell lysates were subjected to SDS-PAGE (8%–12%) and then transferred onto the nitrocellulose membranes (Millipore, Billerica, MA). Membranes were blocked in TBST buffer containing 5% bovine serum albumin for 1 h at room temperature and probed with the indicated antibodies at 4 °C overnight. Detection was performed using fluorescently labeled secondary antibodies. The following antibodies were used: HOXC10 (ab153904) obtained from Abcam (Cambridge, MA, USA). NOD1 (A1246) obtained from ABclonal (Wuhan, China). Cleaved PARP (#5625), PCNA (#13110), p-ERK1/2^T202/Y204^ (#4370), ERK1/2 (#9102), p-P38^Thr180/Tyr182^ (#4511), P38 (#8690), p-P65^Ser536^ (#3033), P65 (#8242), p-STAT3^Tyr705^ (#73533), p-STAT3^Ser727^ (#49081), STAT3 (#12640), GPX4 (#59735), and GAPDH (#5174) purchased from Cell Signaling Technology (Danvers, MA, USA).

### Bioinformatics

RNA-Seq datasets for TCGA tumor-normal matched dataset were downloaded using the R package.^60^ The mutation status was inferred from (https://portal.gdc.cancer.gov/). *P* value for each gene pair in the lung cancer patient dataset were computed. For the survival analysis, a publicly available cohort dataset was used.

### RNA interference

Cells were seeded at about 40% confluence on 6-well plates. Cells were then transfected with 20 nmol/L siRNAs using lipofectamine^TM^ 2000 transfection reagent (Thermo Fisher Scientific, Waltham, MA). The medium was refreshed 8 h after transfection, and transfection efficiency was assessed by qPCR or Western blotting assays. The HOXC10-siRNA 1# target sequence was 5′- GCTGGAATTGGAGAAAGAA-3′. The HOXC10-siRNA 2# target sequence was 5′-CAGACGCTGGAATTGGAGA-3′. The indicated siRNAs were synthesized by GenePharma (Shanghai, China).

### Plasmids and cloning

Human HOXC10 cDNA was amplified by PCR using complementary DNA derived from 293 T cells. The DNA fragments were cloned into the expression vector pcDNA3.1 vector (Invitrogen) using the Gibson assembly kit (New England Biolabs). HOXC10 shRNA cloned in plKO.1 vector according to the Quik-Change Directed mutagenesis kit (Stratagene, La Jolla, CA). The HOXC10-shRNA 1# target sequence was 5′-TCTCCAATTCCAGCGTCTG-3′. The HOXC10-shRNA 2# target sequence was 5′-CAGACGCTGGAATTGGAGA-3′. Lentivirus was packaged by co-transfection with the envelope plasmid pMD2.G, the packaging plasmid psPAX2, and the shRNA sequence into 293 T cells. Cells infected with the virus and selected by 1 μg/mL puromycin (YEASEN, Shanghai, China).

### Luciferase activity assay

*NOD1* DNA insert sequences were amplified from 293 T by PCR assays. Purified insert DNAs were then subcloned into a pGL3 vector (Promega). The following luciferase assay were performed on H441-BM cells, transfected using the Lipofectamine^TM2000^ reagent (Thermo Fisher Scientific) for 48 h. Finally, cells were lysed in 50 μL 1 × passive lysis buffe (Promega) for storing at −80 °C. The luciferase expression was detected by the Varioskan Flash Multimode Reader (Thermo Scientific).

### Osteoclastogenesis assay

In total, 1 × 10^6^ Murine BMMs cells were cultured in complete α-MEM supplemented with 25 ng/mL RANKL (Peprotech, Rocky Hill, NJ, USA) and 50 ng/mL M-CSF in the presence of cell medium. The leukocyte acid phosphatase kit (Sigma-Aldrich, St. Louis, MO, USA) was used for TRAP staining on day 4. The multinucleated TRAP^+^ positive cells (mature osteoclasts) were monitored by a Leica microscope (Leica, DM4000b).

### RNA-sequencing

Total RNA was extracted using RNAiso plus (TaKaRa Biotechnology). Library preparation and sequencing were performed by Shanghai Majorbio Bio-pharm Biotechnology Co., Ltd. (Shanghai, China). Library preparation and barcoding was performed using the TruSeqTM RNA sample preparation Kit (CA, USA) according to the manufacturer’s protocol. An average of 46 million paired reads was sequenced by Illumina Novaseq 6000 platform (Illumina, CA, USA). Differential expression genes (DEGs) were performed according to the fragments per kilobase of exon per million mapped reads (FPKM) method. Differentially expressed genes (*P*adjust < 0.05) were subjected to KEGG analysis.

### Quantitative real-time PCR

RNA extraction was using the RNA TRIzol Reagent (Invitrogen, Eugene, OR). RNA samples (1 μg) were reverse-transcribed using the PrimeScript RT reagent kit (TaKaRa Biotechnology, Dalian, China). Quantitative real-time PCR (qPCR) was performed using a Power SYBR® Premix Ex Taq kit (TaKaRa Biotechnology) according to manufacturer’s instructions. The mRNA expression of indicated genes was quantified by 2^-ΔΔCt^ method and normalized to the expression of GAPDH. The sequence of forward and reverse primers is listed in Table S1.

### ChIP-qPCR assay

The SimpleChIP^®^ Plus Enzymatic Chromatin Immunoprecipitation Kit (#9004, CST, MA, USA) was used for the chromatin Immunoprecipitation (ChIP) assay. Cells were were fixed with 1% formaldehyde for 15 min at room temperature and Cross-linking was quenched with Glycerin. An average chromatin fragment size of 300–1 000 bp generated by digesting for 30 min. These chromatin samples were incubated overnight with FLAG antibodies (anti-FLAG, MBL), which were retrieved with Protein A/G Dynabeads (bimake, TX, USA). The indicated primers are listed in Table S1.

### Electrophoretic mobility shift assay

In total, 50 nmol/L FAM-labeled-DNA was pre-incubated with 400 ng NOD1 protein in reaction buffer (100 mmol/L NaCl, 8% glycerol, 20 mmol/L HEPES, pH 7.5, and 1 mmol/L DTT) for 20 min. The samples resolved by 10% polyacrylamide gel electrophoresis in 0.5×Tris-borate-EDTA buffer (100 V) at 4 °C for 1 h. Images were acquired using the Tanon-5200 chemiluminescent imaging system (Tanon Science & Technology Co., Ltd.). DNA probes were shown in Table S2.

### Clonogenic assay

Cells were plated in 12-well plates at 2 000 cells/well and treated with the indicated agents next day. Cells were then cultured with indicated treatment in complete media for 10 days. Cells in plates were fixed with methanol and then stained with crystal violet. Crystal violet was dissolved in 10% acetic acid and measured at 595 nm. The average OD_595_ of untreated cells was set to 100%, and the percentage of treatment group cells were calculated accordingly.

### Cell viability assay

Cells were plated in 96-well plates at 3 000 cells/well and treated with the indicated agents next day. Cell viability was measured using a Cell Counting Kit-8 (CCK-8, Abcam). The average OD_450_ of untreated cells was set to 100%, and the percentage of viable cells were calculated accordingly. The half inhibitory concentration (IC_50_) values were calculated with the Graphpad software. Cells were treated with MEK162, TTI-101 or combinations for 48 h to analyze the drug synergistic effect. The value combination index (CI) was calculated using the CalcuSyn software. CI < 1 were considered synergistic.

### EdU incorporation assay

Cells were seeded into 96 well plates. After virous treatments, cells were labeled with 10 mmol/L EdU (ThermoFisher Scientific) over 4 h, collected by trypsinization and fixed (4% PFA) according to the manufacturer’s instructions. Data were recorded by Data were recorded by a luminescent plate reader.

### Transwell assay

Cells were seeded on the transwell inserts (8.0 μm pore size, Corning, NY, USA) at (2 × 10^4^) cells/well and cultured in medium containing 10% FBS for 72 h. Migrated cells in transwell inserts were fixed with methanol and then stained with crystal violet. Crystal violet was dissolved in 10% acetic acid and measured at 595 nm. The average OD_595_ of untreated cells was set to 100%, and the percentage of treatment group cells was calculated accordingly.

### Cell apoptosis assay

Cells were plated in 6-well plates at 1 × 10^5^ cells/well and treated with the indicated agents next day for 48 or 72 h. Cells were washed with cold PBS and resuspended in Annexin V binding buffer and stained with Annexin V and PI at room temperature by using the Annexin V Apoptosis Detection Kit (BD Biosciences, San Jose, CA, USA). Annexin V-positive cells were detected using BD FACSCanto II (BD Biosciences) within 0.5 h after staining. 1 × 10^4^ cells were collected and analyzed with FlowJo version 7 software module.

### ROS measurement

Cells were plated in 6-well plates at 1 × 10^5^ cells/well and treated with the indicated agents next day for 72 h. Cells were washed with cold PBS and and stained with DCFH-DA (25 μmol/L; Sigma, St. Louis, MO) at room temperature for 30 min. DCFH-DA-positive cells were detected using BD FACSCanto II (BD Biosciences) within 0.5 h after staining. 1 × 10^4^ cells were collected and analyzed with FlowJo version 7 software module.

### Glutathione detection

Cells were plated in 6-well plates at 5 × 10^5^ cells/well and treated with the indicated agents next day for 72 h. GSH and GSSG levels were analyzed using a GSH/GSSG assay kit (Promega) according to the manufacturer’s instructions. GSH and GSSG concentration was analyzed from the internal standard curve and normalized t untreated cells plates.

### BODIPY-C11 assay

The lipid reactive oxygen species (ROS) level was measured using the BODIPY-C11 dye (Thermo Fisher Scientific) on a 96 well plate reader. In brief, cells were seeded into 6-well plates (4 × 10^4^ cells/well) and then stained with 2.5 μmol/L BODIPY-C11 for 30 min at 37 °C. After washing with phosphate buffered saline (PBS) to remove the unincorporated dye, the stained cells were detected by a 96 well plate reader. The relative lipid ROS level was analyzed.

### Hematoxylin-Eosin (H&E) staining

Deparaffinized tumor tissue sections were stained with hematoxylin for 5 min, separated by hydrochloric acid and ethanol for 30 s, flushed with distilled water for 15 min, and airdried. Stained with eosin for 1 min and air-dried, sealed with neutral gel and observed under a microscope.

## Animal experiment

### Left ventricle injections

On the day of injection, H441 and H441 (Ctrl and shHOXC10) cells were trypsinized, collected, and resuspended at 1 × 10^6^ cells/mL in ice-cold phosphate-buffered saline (PBS; without calcium or magnesium). After the mouse was anesthetized, it was in the supine position and followed by cleaning the thoracic area with an alcohol swab. Using a 27-gauge needle, we injected 100 μL of the cancer cell suspension (1 × 10^5^ cancer cells per animal) into the left cardiac ventricle of the mouse (6-week-old BALB/c nude mice). The mouse then recovered on a heating pad.^61^ Following IC injection of cancer cells, mice were maintained under pathogen-free conditions. Two weeks postinjection, the mice were sacrificed. We then extracted their femurs and tibiae and maintained them in culture as described above. At this time point, the left ventricle injected mice began to look unhealthy and/or in pain. Per University policy and humane treatment of the animal subjects for research, the animals were sacrificed to avoid suffering.

### Drug treatment

The mice were treated with TTI-101 (25 mg/kg) or MEK162 (25 mg/kg) by intraperitoneal injection (i.p.) for 4 weeks, beginning 7 days after cancer cells injection. Bioluminescence images (BLI) were quantified after intraperitoneal injection of D-luciferin potassium (Beyotime, Shanghai, China) into the mice (50 mg/kg) by using the Xenogen IVIS system (Caliper Life Sciences, Hopkinton, MA, USA). The osteolytic status of the spine was scanned by micro-CT (Skyscan 1272, Bruker, 60 kV, 166 µA, 9 µm). Region-of-interest (ROI) was analyzed (0.215 mm,12 image slices to 1.72 mm, 106 image slices) by using CTAo1 software (Bruker microCT).

### Mini-PDX model

The in vivo pharmacological tests were conducted using a mini-patient-derived xenograft (mini-PDX) model (LIDE Biotech, Shanghai, China).^26,62^ Briefly, fresh tumor specimens were acquired from a male lung cancer with bone metastasis patient at Fudan University Shanghai Cancer Center (FUSCC). The tumor tissue was digested with collagenase for 1 h at 37 °C. After removal of blood cells and fibroblasts with magnetic beads, bone metastasis tumor cells were collected to fill OncoVee capsules (LIDE Biotech, Shanghai, China, 2 000 cells). Capsules were implanted into BALB/c nude mice (female, 5 weeks, 3 capsules per mouse). The mice with capsules were treated with TTI-101 (25 mg/kg) or MEK162 (25 mg/kg) by intraperitoneal injection (i.p.) for 7 continuous days. Finally, each capsule was removed from mice and measured cell viability according to the relative luminance unit (RLU) using a CellTiter-Glo Luminescent Assay (Promega). Relative viability = (RLU of treatment D7- RLU of bassline) / (RLU of vehicle D7- RLU of bassline) ×100. The clinical lung cancer bone metastasis sample was approved by the FUSCC Ethics Committee (number: 050432-4-2108*). The protocol of mini-PDX was approved by the Shanghai LIDE Biotech Ethics Committee (number: LWIACUC002).

### Study approval

The Institutional Animal Care and Use Committee of Fudan University Shanghai Cancer Center approved the use of animal models in this study.

### Statistical analysis

Data are presented as the mean ± sem. Statistical significance was determined by two-tailed unpaired *t* test, one-way ANOVA comparison test and log-rank test, as appropriate. The specific tests are described in the Figure legends. Statistical tests were performed using GraphPad Prism (version 8.0).

### Supplementary information

Supplementary information
