# Supplementary material for: The HOXC10/NOD1/ERK axis drives osteolytic bone metastasis of pan-KRAS-mutant lung cancer
Source: Bone Res. 2024 Aug 27;12:47. doi: 10.1038/s41413-024-00350-8 (PMC11349752; doi:10.1038/s41413-024-00350-8)
Supplement: Supplementary file 1 — Supplementary information [file 41413_2024_350_MOESM1_ESM.docx]

**Supplemental Data**

**1. Figure S1.** Reducing HOXC10 expression inhibits cancer cell growth capacity in KRAS-mutant lung cancer cells *in vitro*.

**2. Figure S2.** Knockdown efficiency of siRNAs that target gene by immunoblot analysis.

**3. Figure S3.** HOXC10 inhibition plus TTI-001 induces osteoclastogenesis.

**4. Figure S4.** HOXC10 inhibition and TTI-101 combination induces cell apoptosis and impairs cell growth in KRAS-mutant lung cancer cells.

**5. Figure S5.** HOXC10 and STAT3 co-inhibition stimulates ferroptosis.

**6. Figure S6.** MEK162 plus TTI-101 combination selectively killed lung cancer bone metastasis cells.

**7. Figure S7.** MEK162 and TTI-101 combination has little toxic effects on mouse body weight and blood biochemistry parameters.


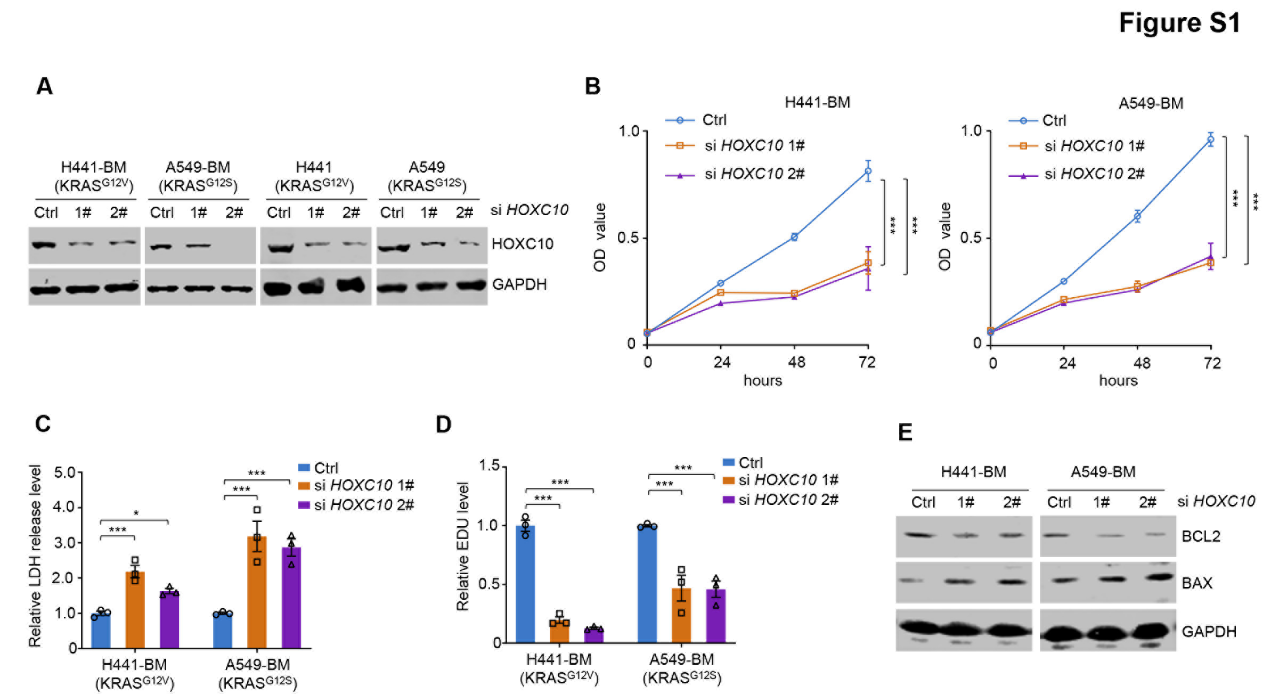


**Figure S1: Reducing HOXC10 expression inhibits cancer cell growth capacity in KRAS-mutant lung cancer cells *in vitro.* A.** Cells were transfected with 20 nM siRNAs targeting HOXC10 for 48 hours and HOXC10 protein expression levels were detected by Western blotting assays. **B.** Cell viability was measured using a Cell Counting Kit-8 (CCK-8) at indicated time points. Relative cell viability (normalized to day 0) is plotted. Data represent the mean ± s.e.m.. Data were performed one-way ANOVA with Tukey’s multiple comparison test, ****P* < 0.001. **C-D.** Cells were transfected with 20 nM siRNAs targeting HOXC10 for 72 hours and LDH **(C)** and EDU **(D)** levels were detected by commercial kits. Data represent the mean ± s.e.m.. Data were performed one-way ANOVA with Tukey’s multiple comparison test, **P* < 0.05, ****P* < 0.001. **E.** Cells were transfected with 20 nM siRNAs targeting HOXC10 for 48 hours and BCL2 and BAX protein expression levels were detected by Western blotting assays. Data in B, C and D represent three technical replicates, representative of three independent experiments with similar results.


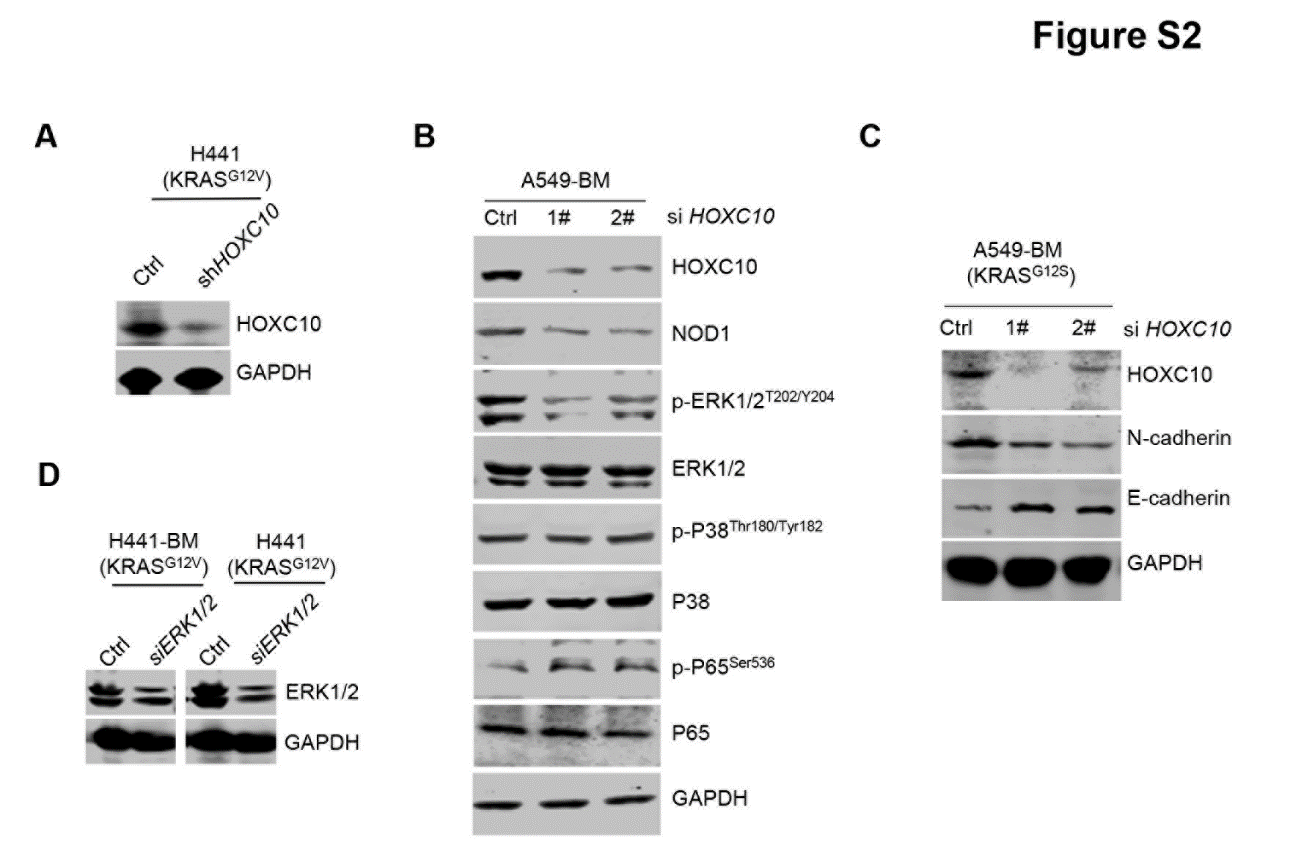


**Figure S2: Knockdown efficiency of siRNAs that target gene by immunoblot analysis. A.** Cells were transfected with 20 nM siRNAs targeting HOXC10 for 48 hours and HOXC10 protein expression levels were detected by Western blotting assays. **B.** *HOXC10* knockdown reduced NOD1 and p-ERK1/2^T202/Y204^ expression but not p-P38^Thr180/Tyr182^ and p-P65^Ser536^ **C.** Cells transfected with the indicated siRNAs for 48 hours The level of E-cadherin and N-cadherin was examined by western blot assay. **D.** Cells were transfected with 20 nM siRNAs targeting ERK1/2 for 48 hours and ERK1/2 protein expression levels were detected by Western blotting assays.


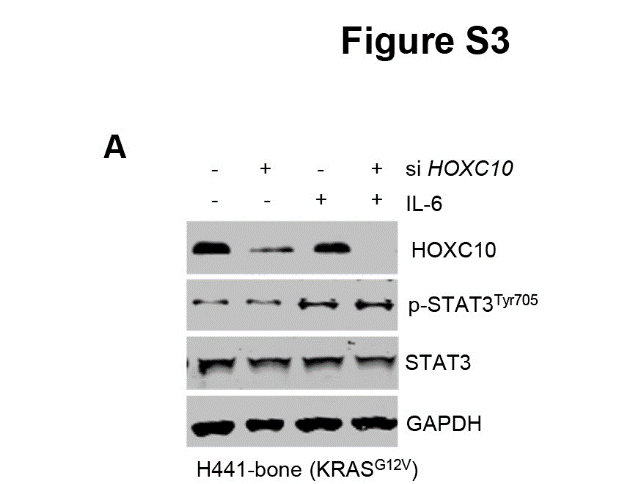


**Figure S3: HOXC10 inhibition plus TTI-001 induces osteoclastogenesis. A.** HOXC10, p-STAT3^Tyr705^ and STAT3 protein levels in H441-bone cells. Cells were transfected with 20 nM siRNAs targeting HOXC10 and treated with or without IL-6 (20 ng/mL) for 48 hours. The immunoblots were contemporaneous and run in parallel from the same biological replicate, representative of three independent experiments.


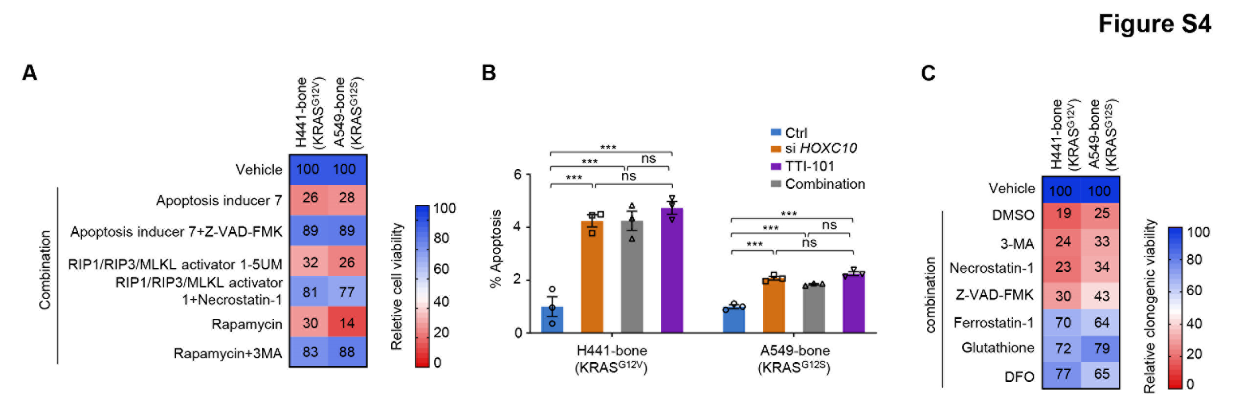


**Figure S4: HOXC10 inhibition and TTI-101 combination induces cell apoptosis and impairs cell growth in KRAS-mutant lung cancer cells. A.** Rescue of cell death induced by the indicated death inducers and corresponding inhibitors. Apoptosis was induced by Apoptosis inducer 7 (1 μM for 6 hours) and rescued by treatment with caspase inhibitor (Z-VAD-FMK,10 µM). Necrosis was induced by treatment with RIP1/RIP3/MLKL activator 1 (5 μM for 6 hours) and rescued by treatment with Necrostatin-1 (20 μM). Autophagy was induced by Rapamycin (200 nM for 6 hours) and rescued by treatment with 3-MA (1mM). Cells were then cultured for an additional 72 hours. **B.** Apoptosis of indicated cells were detected by flow cytometry. Treated cells were cultured for 72 hours. The apoptosis cells are calculated by normalizing the untreated group as 100%. Data were performed one-way ANOVA with Tukey’s multiple comparison test and shown as mean ± s.e.m.; ****P* < 0.001. **C.** Ferroptosis inhibitors rescue cell death induced by HOXC10 inhibition and TTI-101 combination. Cells with or without stable knockdown of HOXC10 were pretreated with HOXC10 TTI-101 for 12 hour and then treated the indicated cell death inhibitors (3-MA-1 mM, Necrostatin-1-20 μM, Z-VAD-FMK-10 μM, Ferrostatin-1-2 μM, Glutathione-1 mM, Deferoxamine (DFO)-100 μM) for an additional 72 hours. Treated cells were seeded in six-well plates, cultured for 7 days, and stained with crystal violet. The relative clonogenic viability is calculated by normalizing the untreated group as 100%.


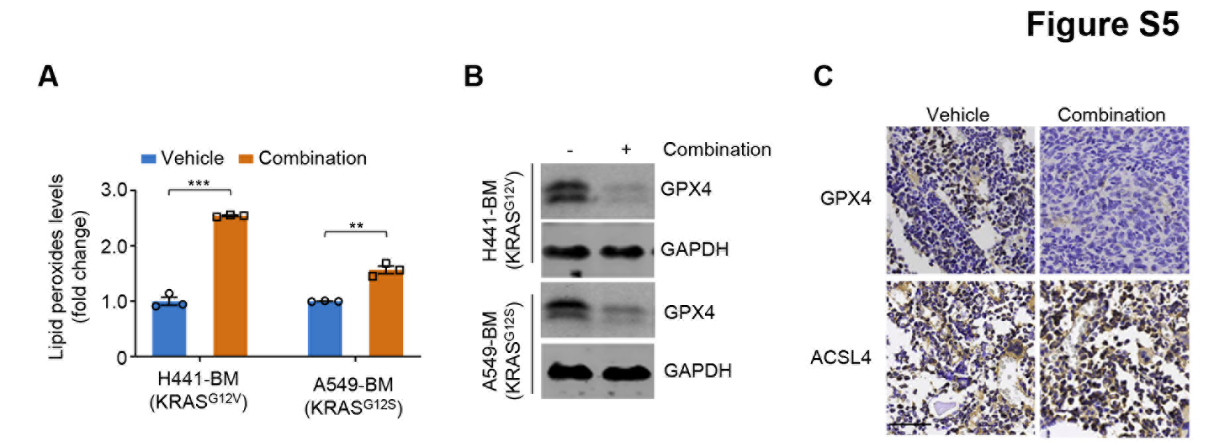


**Figure S5: HOXC10 and STAT3 co-inhibition stimulates ferroptosis. A.** Cells were treated with HOXC10 inhibition and TTI-101 (4 μM) combination for 72 hours. The lipid ROS level was evaluated by BODIPYC11 dye on a luminescent plate reader. **B.** Cells were treated with HOXC10 inhibition and TTI-101 (4 μM) combination for 72 hours. GPX4 protein expression levels were detected by Western blotting assays. Data were performed unpaired two-sided Student’s *t*-test, ***P* < 0.01, ****P* < 0.001. **C.** After 7 days intracardiac injection, mice were treated with TTI-101 (25 mg/kg) for an additional 28 days. Representative images of immunohistochemical (IHC) staining. Tumor tissue on day 35 were examined for GPX4 and ACSL4 expression. Scale, 25 μm. Data in A represent three technical replicates, representative of three independent experiments with similar results.


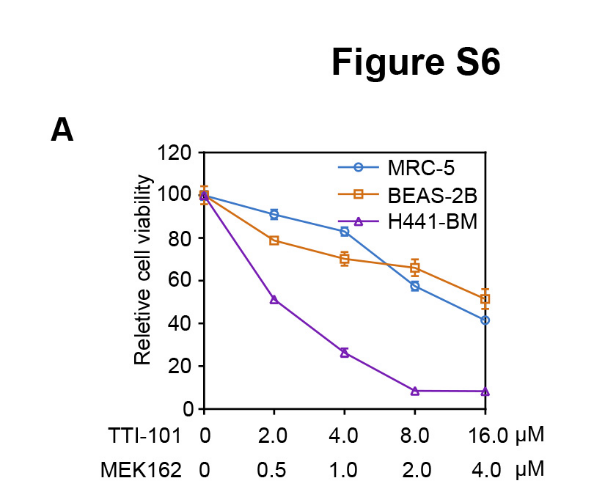


**Figure S6: MEK162 plus TTI-101 combination selectively killed lung cancer bone metastasis cells. A.** Synergistic interaction between MEK162 and TTI-101 in metastatic lung cancer cells (H441-BM) and normal lung epithelial cells (MRC-5 and BEAS-2B). Cells were treated with various concentrations of indicated inhibitors for 72 hours. Data in A represent three technical replicates, representative of three independent experiments with similar results.


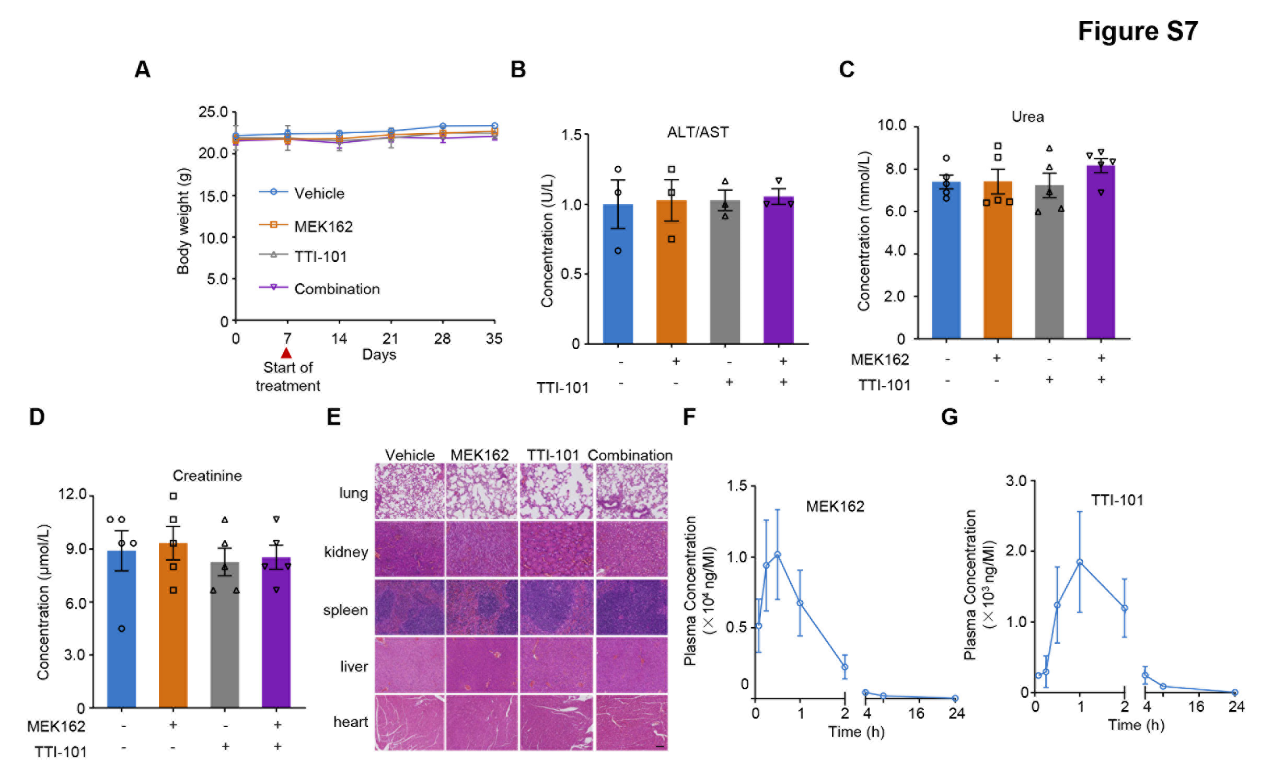


**Figure S7: MEK162 and TTI-101 combination has little toxic effects on mouse body weight and blood biochemistry parameters. A.** The mouse body weight (*n* = 6). Data are expressed as mean ± s.e.m. **B-D.** The serum biochemical testing of mice in mouse model (*n* = 3). Data are expressed as mean ± s.e.m. Alanine aminotransferase, ALT; Aspartate aminotransferase, AST. n = 3 per group in **B.** n = 5 per group in **C** and **D.** **E.** H&E staining was used to detect the changes in the important organs. Scale, 50 μm. n = 3 per group. **F-G.** Plasma concentration–time curves of multiple components of MEK162 (25 mg/kg, in **F**) and TTI-101 (25 mg/kg, in **G**) in mice. n = 4 per group.

**Supplemental Table**

**Supplemental Table 1** Primers used for PCR assays, related to Figure 1,4 and Supplemental Figure 3

**Supplemental Table 2** DNA probes for EMSA, related to Figure 4

**Supplementary Table 3** MEK 162 TD Result, related to Supplemental Figure 7

**Supplementary Table 4** TTI-101 TD Result, related to Supplemental Figure 7

**Supplementary Table 5** MEK162 Plasma Result, related to Supplemental Figure 7

**Supplementary Table 6** TTI-101 Plasma Result, related to Supplemental Figure 7

**Supplementary Table 1** Primers used for PCR assays

|  | | |  |
| --- | --- | --- | --- |
| Application | Classification | Gene symbol | Sequence (5'-3’) |
| qPCR | HOXC10 and its target genes | *HOXC10* Forward | CTATCCGTCCTACCTCTCGCA |
|  |  | *HOXC10* Reverse | ACATGCAGCAGACATTCTCCT |
|  |  | *NOD1* Forward | GATTGGAGACGAAGGGGCAA |
|  |  | *NOD1* Reverse | TGGCTGTGTTCTTCTGCAGT |
|  |  | *TAB3* Forward | CCTGAAATTCCAGAGGGCGT |
|  |  | TAB3 Reverse | TGCTGCATGCTGAGGATCAA |
|  |  | *IL6* Forward | TGCGTCCGTAGTTTCCTTCT |
|  |  | IL6 Reverse | GCCTCAGACATCTCCAGTCC |
|  | GAPDH | *GAPDH* Forward | AATCCCATCACCATCTTCCA |
|  |  | *GAPDH* Reverse | TGGACTCCACGACGTACTCA |
| ChIP-qPCR | *NOD1* promoter region | *NOD1* Forward | AGATTAGGAAGGGACCAATGTGT |
|  |  | *NOD1* Reverse | ACTCAACAATGGTTCCAAATAGCC |

**Supplementary Table 2** DNA probes for EMSA

| Classification | probe symbol | Sequence (5'-3’) |
| --- | --- | --- |
| *NOD1* probe (WT) | sense | CGTGTCCGTGTGAAGAGACCACCAAAAGGCTTTGCGTGAGCAA**TAAA**GCTGTTTAT |
|  | anti-sense | ATAAACAGCTTTATTGCTCACGCAAAGCCTTTTGGTGGTCTCTTCACACGGACACG |
| *NOD1* probe (Mut) | sense | CGTGTCCGTGTGAAGAGACCACCAAAAGGCTTTGCGTGAGCAA**CGCG**GCTGTTTAT |
|  | anti-sense | ATAAACAGCTTCGCGGCTCACGCAAAGCCTTTTGGTGGTCTCTTCACACGGACACG |
| The probes used in the EMSA analysis are shown in the table with the depletion of substitution of mutated bases highlighted in red. | | |

**Supplementary Table 3** MEK 162 TD Result

|  | **Time (hr)** | **Matrix** | **Mouse #** | **Tissue weight (g)** | **Volume of 50% MeOH in ACN for Homogenizing (mL)** | **Concentration in Tissue Homogenate (ng/mL)** | | | | **Concentration in Tissue (ng/g)** | | | |
| --- | --- | --- | --- | --- | --- | --- | --- | --- | --- | --- | --- | --- | --- |
|  |  |  |  |  |  | **Individual** | **Mean** | **SD** | **CV(%)** | **Individual** | **Mean** | **SD** | **CV(%)** |
| MEK162 at 25 mg/kg | 1 | Heart | Mouse #1 | 0.107 | 0.427 | 164 | 235 | 136 | 57.7 | 819 | 1174 | 678 | 57.7 |
|  |  |  | Mouse #2 | 0.105 | 0.420 | 333 |  |  |  | 1664 |  |  |  |
|  |  |  | Mouse #3 | 0.071 | 0.285 | 363 |  |  |  | 1813 |  |  |  |
|  |  |  | Mouse #4 | 0.102 | 0.407 | 79.5 |  |  |  | 398 |  |  |  |
|  |  | Liver | Mouse #1 | 0.149 | 0.597 | 159 | 458 | 259 | 56.4 | 796 | 2292 | 1293 | 56.4 |
|  |  |  | Mouse #2 | 0.112 | 0.449 | 505 |  |  |  | 2525 |  |  |  |
|  |  |  | Mouse #3 | 0.185 | 0.738 | 388 |  |  |  | 1941 |  |  |  |
|  |  |  | Mouse #4 | 0.128 | 0.511 | 781 |  |  |  | 3905 |  |  |  |
|  |  | Spleen | Mouse #1 | 0.135 | 0.541 | 685 | 1149 | 951 | 82.8 | 3424 | 5744 | 4754 | 82.8 |
|  |  |  | Mouse #2 | 0.109 | 0.435 | 53 |  |  |  | 264 |  |  |  |
|  |  |  | Mouse #3 | 0.103 | 0.410 | 2126 |  |  |  | 10632 |  |  |  |
|  |  |  | Mouse #4 | 0.101 | 0.403 | 1731 |  |  |  | 8657 |  |  |  |
|  |  | Lung | Mouse #1 | 0.088 | 0.353 | 170 | 250 | 132 | 52.8 | 848 | 1251 | 660 | 52.8 |
|  |  |  | Mouse #2 | 0.105 | 0.421 | 346 |  |  |  | 1730 |  |  |  |
|  |  |  | Mouse #3 | 0.091 | 0.364 | 378 |  |  |  | 1890 |  |  |  |
|  |  |  | Mouse #4 | 0.095 | 0.378 | 108 |  |  |  | 538 |  |  |  |
|  |  | Kidney | Mouse #1 | 0.124 | 0.495 | 1058 | 799 | 433 | 54.2 | 5291 | 3996 | 2167 | 54.2 |
|  |  |  | Mouse #2 | 0.128 | 0.513 | 1029 |  |  |  | 5144 |  |  |  |
|  |  |  | Mouse #3 | 0.115 | 0.460 | 152 |  |  |  | 761 |  |  |  |
|  |  |  | Mouse #4 | 0.181 | 0.723 | 957 |  |  |  | 4787 |  |  |  |
|  |  | Tumor | Mouse #1 | 0.208 | 0.832 | 319 | 200 | 139 | 69.3 | 1595 | 999 | 693 | 69.3 |
|  |  |  | Mouse #2 | 0.072 | 0.288 | 316 |  |  |  | 1581 |  |  |  |
|  |  |  | Mouse #3 | 0.102 | 0.408 | 49.2 |  |  |  | 246 |  |  |  |
|  |  |  | Mouse #4 | 0.093 | 0.371 | 115 |  |  |  | 575 |  |  |  |

**Supplementary Table 4** TTI-101 TD Result

| **Dose** | **Time (hr)** | **Matrix** | **Mouse #** | **Tissue weight (g)** | **Volume of 50% MeOH in ACN for Homogenizing (mL)** | **Concentration in Tissue Homogenate (ng/mL)** | | | | **Concentration in Tissue (ng/g)** | | | |
| --- | --- | --- | --- | --- | --- | --- | --- | --- | --- | --- | --- | --- | --- |
|  |  |  |  |  |  | **Individual** | **Mean** | **SD** | **CV(%)** | **Individual** | **Mean** | **SD** | **CV(%)** |
| TTI-101  at 25 mg/kg | 1 | Heart | Mouse #1 | 0.101 | 0.404 | 3255 | 2805 | 1537 | 54.8 | 16276 | 14025 | 7685 | 54.8 |
|  |  |  | Mouse #2 | 0.062 | 0.246 | 3965 |  |  |  | 19825 |  |  |  |
|  |  |  | Mouse #3 | 0.080 | 0.320 | 543 |  |  |  | 2717 |  |  |  |
|  |  |  | Mouse #4 | 0.062 | 0.247 | 3456 |  |  |  | 17280 |  |  |  |
|  |  | Liver | Mouse #1 | 0.207 | 0.827 | 10462 | 8702 | 4914 | 56.5 | 52309 | 43508 | 24570 | 56.5 |
|  |  |  | Mouse #2 | 0.084 | 0.335 | 13047 |  |  |  | 65234 |  |  |  |
|  |  |  | Mouse #3 | 0.205 | 0.819 | 1660 |  |  |  | 8299 |  |  |  |
|  |  |  | Mouse #4 | 0.145 | 0.579 | 9638 |  |  |  | 48189 |  |  |  |
|  |  | Spleen | Mouse #1 | 0.213 | 0.852 | 574 | 6711 | 7437 | 111 | 2872 | 33554 | 37185 | 111 |
|  |  |  | Mouse #2 | 0.061 | 0.242 | 4907 |  |  |  | 24535 |  |  |  |
|  |  |  | Mouse #3 | 0.165 | 0.659 | 17518 |  |  |  | 87590 |  |  |  |
|  |  |  | Mouse #4 | 0.117 | 0.467 | 3844 |  |  |  | 19218 |  |  |  |
|  |  | Lung | Mouse #1 | 0.118 | 0.474 | 934 | 3573 | 2066 | 57.8 | 4670 | 17866 | 10328 | 57.8 |
|  |  |  | Mouse #2 | 0.052 | 0.208 | 5967 |  |  |  | 29837 |  |  |  |
|  |  |  | Mouse #3 | 0.076 | 0.302 | 3885 |  |  |  | 19427 |  |  |  |
|  |  |  | Mouse #4 | 0.102 | 0.406 | 3506 |  |  |  | 17532 |  |  |  |
|  |  | Kidney | Mouse #1 | 0.136 | 0.542 | 762 | 4493 | 2623 | 58.4 | 3812 | 22464 | 13117 | 58.4 |
|  |  |  | Mouse #2 | 0.116 | 0.465 | 5808 |  |  |  | 29041 |  |  |  |
|  |  |  | Mouse #3 | 0.124 | 0.494 | 4680 |  |  |  | 23402 |  |  |  |
|  |  |  | Mouse #4 | 0.094 | 0.376 | 6721 |  |  |  | 33604 |  |  |  |
|  |  | Tumor | Mouse #1 | 0.186 | 0.742 | 101 | 704 | 550 | 78.0 | 506 | 3522 | 2749 | 78.0 |
|  |  |  | Mouse #2 | 0.081 | 0.323 | 868 |  |  |  | 4342 |  |  |  |
|  |  |  | Mouse #3 | 0.108 | 0.430 | 466 |  |  |  | 2331 |  |  |  |
|  |  |  | Mouse #4 | 0.103 | 0.412 | 1382 |  |  |  | 6911 |  |  |  |

**Supplementary Table 5** MEK162 Plasma Result

| **Information/Parameter** | **PK Parameters** | | | | **N** | **Mean** | **SD** | **CV(%)** |
| --- | --- | --- | --- | --- | --- | --- | --- | --- |
| **Animal** | Mouse#1 | Mouse#2 | Mouse#3 | Mouse#4 |  |  |  |  |
| **Analyte** | / | | | |  |  |  |  |
| **Route of Administration** | IP | | | |  |  |  |  |
| **Dosage** | 25 | mg/kg | | |  |  |  |  |
| **Rsq_adjusted** | 1.00 | 0.920 | 0.942 | 0.973 | NA | NA | NA | NA |
| **No_points_lambda_z** | 3 | 3 | 3 | 3 | NA | NA | NA | NA |
| **T_1/2_ (h)** | 1.22 | 6.32 | 6.79 | 3.12 | 3 | 4.36 | 2.65 | 60.8 |
| **T_max_ (h)** | 0.25 | 0.50 | 0.50 | 0.25 | 3 | 0.375 | 0.144 | 38.5 |
| **C_max_ (ng/mL)** | 9821 | 5815 | 19358 | 6258 | 3 | 10313 | 6291 | 61.0 |
| **AUC_last_ (h·ng/mL)** | 13951 | 10772 | 38033 | 10762 | 3 | 18379 | 13188 | 71.8 |
| **AUC_inf_ (h·ng/mL)** | 14045 | 11043 | 38904 | 10775 | 3 | 18692 | 13556 | 72.5 |
| **MRT_last_ (h)** | 1.28 | 2.58 | 2.63 | 1.77 | 3 | 2.07 | 0.65 | 31.7 |
| **MRT_inf_ (h)** | 1.34 | 3.33 | 3.33 | 1.80 | 3 | 2.45 | 1.03 | 42.2 |
| **AUC_Extrap_ (%)** | 0.671 | 2.46 | 2.24 | 0.127 | 3 | 1.37 | 1.15 | 83.8 |
| **AUMC_Extrap_ (%)** | 4.88 | 24.4 | 22.7 | 2.00 | 3 | 13.5 | 11.7 | 86.6 |

**Supplementary Table 6** TTI-101 Plasma Result

| **Information/Parameter** | **PK Parameters** | | | | **N** | **Mean** | **SD** | **CV(%)** |
| --- | --- | --- | --- | --- | --- | --- | --- | --- |
| **Animal** | Mouse#1 | Mouse#2 | Mouse#3 | Mouse#4 |  |  |  |  |
| **Analyte** | / | | | |  |  |  |  |
| **Route of Administration** | IP | | | |  |  |  |  |
| **Dosage** | 25 | mg/kg | | |  |  |  |  |
| **Rsq_adjusted** | 0.998 | 0.412 | 0.930 | 0.938 | NA | NA | NA | NA |
| **No_points_lambda_z** | 3 | 3 | 3 | 4 | NA | NA | NA | NA |
| **T_1/2_ (h)** | 3.19 | 4.33 | 4.11 | 2.93 | 3 | 3.64 | 0.68 | 18.7 |
| **T_max_ (h)** | 1.00 | 1.00 | 0.500 | 1.00 | 3 | 0.875 | 0.250 | 28.6 |
| **C_max_ (ng/mL)** | 2688 | 186 | 2031 | 3324 | 3 | 2057 | 1355 | 65.9 |
| **AUC_last_ (h·ng/mL)** | 7703 | 324 | 5126 | 8279 | 3 | 5358 | 3626 | 67.7 |
| **AUC_inf_ (h·ng/mL)** | 7722 | 383 | 5149 | 8304 | 3 | 5389 | 3608 | 66.9 |
| **MRT_last_ (h)** | 3.09 | 2.25 | 2.91 | 2.83 | 3 | 2.77 | 0.36 | 13.1 |
| **MRT_inf_ (h)** | 3.15 | 4.11 | 3.04 | 2.90 | 3 | 3.30 | 0.55 | 16.7 |
| **AUC_Extrap_ (%)** | 0.240 | 15.5 | 0.457 | 0.294 | 3 | 4.13 | 7.59 | 184 |
| **AUMC_Extrap_ (%)** | 2.18 | 53.8 | 4.50 | 2.86 | 3 | 15.8 | 25.3 | 160 |
